# Spatial Mapping of Myeloid Cells and Macrophages by Multiplexed Tissue Staining

**DOI:** 10.3389/fimmu.2018.02925

**Published:** 2018-12-14

**Authors:** Joshua Saylor, Zhaoxuan Ma, Helen S. Goodridge, Fangjin Huang, Anne E. Cress, Stephen J. Pandol, Stephen L. Shiao, Adriana C. Vidal, Lily Wu, Nicholas G. Nickols, Arkadiusz Gertych, Beatrice S. Knudsen

**Affiliations:** ^1^Departments of Biomedical Sciences, Pathology, Surgery and Medicine, Cedars-Sinai Medical Center, Los Angeles, CA, United States; ^2^Molecular and Cellular Biology, University of Arizona Cancer Center, University of Arizona, Tucson, AZ, United States; ^3^Department of Molecular and Medical Pharmacology and Radiation Oncology, David Geffen School of Medicine, University of California, Los Angeles, Los Angeles, CA, United States

**Keywords:** multiplex, immunofluorescence, immunohistochemistry, macrophage, myeloid, spatial profiling, FFPE

## Abstract

An array of phenotypically diverse myeloid cells and macrophages (MC&M) resides in the tumor microenvironment, requiring multiplexed detection systems for visualization. Here we report an automated, multiplexed staining approach, named PLEXODY, that consists of five MC&M-related fluorescently-tagged antibodies (anti - CD68, - CD163, - CD206, - CD11b, and - CD11c), and three chromogenic antibodies, reactive with high- and low-molecular weight cytokeratins and CD3, highlighting tumor regions, benign glands and T cells. The staining prototype and image analysis methods which include a pixel/area-based quantification were developed using tissues from inflamed colon and tonsil and revealed a unique tissue-specific composition of 14 MC&M-associated pixel classes. As a proof-of-principle, PLEXODY was applied to three cases of pancreatic, prostate and renal cancers. Across digital images from these cancer types we observed 10 MC&M-associated pixel classes at frequencies greater than 3%. Cases revealed higher frequencies of single positive compared to multi-color pixels and a high abundance of CD68+/CD163+ and CD68+/CD163+/CD206+ pixels. Significantly more CD68+ and CD163+ vs. CD11b+ and CD11c+ pixels were in direct contact with tumor cells and T cells. While the greatest percentage (~70%) of CD68+ and CD163+ pixels was 0–20 microns away from tumor and T cell borders, CD11b+ and CD11c+ pixels were detected up to 240 microns away from tumor/T cell masks. Together, these data demonstrate significant differences in densities and spatial organization of MC&M-associated pixel classes, but surprising similarities between the three cancer types.

## Introduction

Diverse subsets of myeloid cells and macrophages (MC&M) are observed in tissues, including both resident and recruited MC&M populations (1–3). MC&Ms are tissue-specific and change in response to infection, inflammation and cancer (4). Macrophages are first produced by erythromyeloid progenitors in the yolk sac and populate the tissues of the developing embryo (5). A broader range of myeloid cells subsequently arises from hematopoietic stem cells in the fetal liver, and then in the bone marrow after birth (6). Some MC&M subtypes are short-lived and replaced frequently by newly recruited cells, whereas others can persist in tissues for years and are maintained by self-renewal (7). While the functional programming of MC&M subpopulations is influenced by their origins, they can further differentiate or become activated in tissues in response to organ-specific microenvironmental factors (8). Monocytes recruited to inflamed tissues can for instance become inflammatory macrophages or monocyte-derived dendritic cells (9), and myeloid cells produced in the bone marrow of tumor-bearing mice and humans can colonize the tumor microenvironment (TME) and develop immunosuppressive activities (10). The immune infiltration of cancers consists of a complicated admixture of tissue and organ resident MC&M populations as well as MC populations from the bone marrow whose production, attraction and phenotypes in the TME are influenced by tumor-derived cytokines.

Immunohistochemistry (IHC) and immunofluorescence (IF) can be used to visualize MC&M populations in formalin fixed and paraffin embedded (FFPE) tissue sections based on the expression of cluster definition (CD) antigens. IF is best suited to accurately analyze co-expressed CD markers, while IHC offers greater morphologic and diagnostic accuracy. Phenotypically distinct MC&M subtypes differ in immune function, response to stimuli regulating chemotaxis and differentiation, and cytokine secretion. While links between phenotype, differentiation and functionality have been established for murine macrophages, the human MC&M compartment remains less well understood (11). In the realm of diagnostic pathology, CD68 and CD163 are established markers of macrophages (12). CD68 is expressed by classically activated, inflammatory, M1 macrophages, while CD163 marks alternatively activated, proangiogenic and prooncogenic M2 macrophages (13). CD163+ macrophages in tumor regions are also referred to as tumor associated macrophages (TAM) (14, 15). The observation of CD68 and CD163 expression in TAMs (16) raised the possibility of a dynamic process underlying M1 and M2 polarization (15, 17). Further, the dynamics of macrophage polarization suggest that phenotypic M1/M2 classification with single markers is simplistic and that multiple, functionally distinct subgroups may exist within the CD68+ and CD163+ macrophage populations. Notably, switching TAMs from M2 to M1 represents a key anti-cancer immunotherapeutic treatment strategy (18).

In addition to TAMs, other subtypes of myeloid cells populate the TME. These include: (i) angiopoietin-2 (Ang-2) receptor, Tie2, expressing monocytes (TEM), (ii) myeloid-derived suppressor cells (MDSC), (iii) tumor-associated neutrophils (TAN), and (iv) tumor- associated dendritic cells (TADC) (19). Similar to TAMs, CD11b+ monocytic and granulocytic MDSCs promote metastatic tumor progression and suppress the immune response to the tumor. In contrast, CD11c+ macrophages regulate innate and adaptive immune responses and play a major role in the treatment response to PD-L1 targeting immune checkpoint inhibition (20).

Phenotypic profiling of the tumor immune response provides important information for tumor sub-classification, treatment decisions and clinical outcome prediction (21–24). Thus, it is imperative to have good and reliable tools to detect immune cell subtypes in tumor samples. We took advantage of the TissueFaxs PLUS instrument, which can be used for multi-color tissue cytometry (25). The TissueFaxs allows acquisition of data from up to 10 fluorophores and to gate cells based on marker expression, shape or size characteristics (26). The use of filter cubes enables color separation and simplifies the optimization of multiplex panels that consist of bright and dim antibody signals (27, 28). In addition, improvements in analysis of digital images using masks over cells and regions (29, 30) and advances in open source and commercial image analysis software have opened the door to data extraction from digital images (31). For example, measuring distances between cells in whole slides recently demonstrated how immune checkpoint inhibition changes the organization of T cell subsets in lung cancer, and that distance between T cells and cancer cells was associated with patient outcome (32).

In immunophenotyping studies, CD8+ T cells have been the primary focus, because of their direct cytotoxicity toward cancer cells and cytotoxic activation through PD-1/PD-L1 checkpoint inhibition (33–36). While CD8+ T cells are regulated by MC&Ms, the systematic profiling of and immunophenotyping of MC&Ms by tissue imaging has not advanced as rapidly as that of T cells. To fill the much-needed technological gap, we combined antibodies reactive toward M1 and M2 macrophages, myeloid cells and dendritic cells (CD68, CD163, CD11b, and CD11c, respectively) into one fluorescently-tagged antibody panel (mIF) panel and included CD206 as a functional M2 macrophage marker. Here, we demonstrate the feasibility of an automated staining process for whole slides with eight antibodies. The assay, named PLEXODY, incorporates automated multiplexed IF (mIF) and multiplex IHC (mIHC) tissue staining, digital image co-registration and data extraction and combines the quantitative strength of IF with the diagnostic capacity of IHC. As a proof of principle, we applied the PLEXODY assay to tissues from pancreatic, prostate and renal cancers and investigated its fitness for the analysis of single positive and multi-color pixels, and for measurements of MC&M-associated pixel densities and their proximities to tumor cells and T cells.

## Materials and Methods

### Tissue Resources

Cases for the study were obtained from the pathology archives at Cedars-Sinai Medical Center by an honest broker in the Biobank and Translational Research Core. The link to any information that can be used to identify patients was destroyed and blocks from each case were labeled with the tissue type and a consecutive number. Therefore, the study was considered exempt from IRB oversight by the IRB committee (IRB # Pro00025521). The de-identified formalin fixed and embedded paraffin (FFPE) blocks were transferred to the research core and sectioned by the core personnel. Slides were labeled with the tissue type and a consecutive number. Unstained slides were provided for the project without any accompanying data.

### Immune Cell Phenotyping in FFPE Tissues Using a Multiplex Antibody Format

#### Antibodies

The staining with a sequence of 5 antibodies was completed on the Ventana Discovery Ultra autostainer. The antibodies were CD68 (clone KP-1 mouse mAb, Ventana Medical Systems cat. 790-2931), CD11c (clone EP1347Y rabbit mAb, Abcam cat. ab52632), MRC1 (CD206) (clone CL0387 mouse mAb, Sigma-Aldrich cat. AMAb90746), CD163 (clone MRQ26 mouse mAb, Ventana Medical Systems cat. 760-4437) and CD11b (clone EPR1344 rabbit mAb, Abcam cat. ab133357).

#### Antibody Optimization

Several parameters were optimized for each antibody in the multiplex IF assay. (1) Antibody–fluorophore pairs were determined based on staining intensities of fluorophores and expression levels of respective antigens. The pairs are: CD68–Discovery Cy5 Kit (Ventana Medical Systems cat. 760-238), CD206–Discovery DCC Kit (Ventana Medical Systems cat. 760-240), CD163–Discovery FAM Kit (Ventana Medical Systems cat. 760-243), CD11c–Discovery Red 610 Kit (Ventana Medical Systems cat. 760-245), and CD11b–Discovery Rhodamine 6G Kit (Ventana Medical Systems cat. 760-244). The secondary antibodies were Discovery OmniMap anti-mouse HRP (Ventana Medical Systems cat. 760-4310) and Discovery OmniMap anti-rabbit- HRP (Ventana Medical Systems cat. 760-4311). (2) Antibody dilutions are optimized using IHC. IHC and IF used the amplification system with similar efficiency. This allowed a smooth transition from IHC to fluorescent detection. (3) To determine the sensitivities of antibodies to heat induced epitope retrieval (HIER), staining intensities were compared for one vs. five HIER steps prior to incubation with each antibody on a separate slide. The top 10% of the histogram of staining intensity by an antibody after 1 retrieval was compared to the top 10 percent of the histogram after 5 retrievals. (4) To confirm that antibodies are completely removed through heat denaturation after staining, each antibody was visualized first with the yellow chromogen, denatured, washed away. The slide was then incubated with secondary antibody and red chromogen. Intensities of red pixels were compared in slides without and with removal of the antibody. If an antibody could not be fully removed, such as the pan-CK antibody, it was used last in the staining sequence.

#### Immunofluorescent Staining

The Ventana Discovery Ultra autostainer was used for staining and antigen retrieval was performed on the deparaffinized slides with Cell Conditioning 1 (CC1) solution (Ventana Medical Systems cat. 950-124) for 64 min at 95°C. All antibody incubation steps occurred at 37°C, primary antibodies were incubated for 32 min and secondary antibodies for 12 min. First, slides were blocked with Discovery Inhibitor for 12 min. The CD11b antibody was applied first at a 1:2000 dilution and visualized with the Discovery Rhodamine 6G Kit. Antibody denaturation was performed with Cell Conditioning 2 (CC2) solution (Ventana Medical Systems cat. 950-123) for 8 min at 91°C. Next, the CD206 antibody was applied at a 1:500 dilution and visualized with the Discovery DCC Kit. Antibody denaturation was again performed with CC2 solution for 8 min at 91°C. Next, the CD11c antibody was applied at a 1:500 dilution and visualized with the Discovery Red 610 Kit. Antibody denaturation was again performed with CC2 solution for 8 min at 91°C. Next, the CD68 antibody was applied and visualized with the Discovery Cy5 Kit. Antibody denaturation was again performed with CC2 solution for 8 min at 91°C. Lastly, the CD163 antibody was applied and visualized with the Discovery FAM Kit. The slide was then counterstained with DAPI (0.1 μg/ml, Thermo Fisher Scientific cat. D3571) and cover slipped with ProLong Gold antifade reagent (Life Technologies cat. P36930).

#### Imaging of Fluorescent Slides

Slides stained with the multiplex IF (mIF) panel were imaged with a TissueFaxs whole slide scanning platform (TissueGnostics USA Ltd, Tarzana, CA) equipped with a 20x objective and a scientific-grade 16-bit monochromatic camera (1392 × 1040 pixel). Each fluorophore was measured using a separate filter cube corresponding to its emission wavelength. DAPI (Chroma, cat. 49000), FITC/CY2 (Chroma, cat. 49002), Cy5 (Chroma, cat. 49006), Gold (Chroma, cat. 49304), Red (Chroma, cat. # 49008), and Aqua (Chroma, cat. 49302). The pixel size of the TissueFaxs is 0.3 × 0.3 μm. Scanned regions are saved as tiles in a lossless jpg format.

The IF stain consists of 5 colors + DAPI that are separately acquired by the TissueFaxs and the output is a gray level image for each fluorophore. The areas for analysis were selected by the study pathologist (BSK) using an adjacent hematoxylin and eosin stained slide as a reference. The pathologist's annotation was transferred to the fluorescent preview image in the TissueFaxs software and the regions of interest were selected for high resolution imaging using the software's marking tools.

#### IHC Staining After IF Staining

Coverslips were removed from slides after all fluorescent images were captured and the slides were placed on the Ventana Discovery Ultra autostainer. Reagents were purchased form Ventana Medical Systems unless indicated otherwise. Antibody denaturation was performed on the slides with CC2 solution for 8 min at 91°C. The slides were blocked with Discovery Inhibitor CM (a component of the Discovery ChromoMap DAB kit) for 12 min. All primary and secondary antibody incubations are conducted at 37°C. All primary antibodies were obtained in a prediluted formulation from Ventana and incubated for 32 min. Prediluted secondary antibodies conjugated to haptens (HQ or NP) were incubated for 16 min. The anti-hapten antibodies conjugated to horse radish peroxidase (anti HQ-HRP) or alkaline phosphatase (anti-NP-AP) were incubated for 16 min. The CD3 (clone 2GV6 rabbit mAb, cat. 790-4341), high molecular weight cytokeratin (HMW-CK) (Ventana CONFIRM anti-keratin mouse monoclonal (clone 34ßE12, cat. 790-4373) and low molecular weight cytokeratin 8/18 (LMW-CK) (pre-diluted cytokeratin 8 & 18 (clones B22.1 & B23.1), cat. 760-4344) were used with Discovery Teal HRP Kit (RUO) (cat. 760-247), Discovery Purple Kit (RUO) (cat. 760-229), and Discovery Yellow Kit (RUO) (cat. 760-239) chromogen kits, respectively. The slide was counterstained with Modified Mayer's Hematoxylin (American MasterTech cat. HXMMH100) and cover-slipped with the toluene free mounting medium EcoMount (BIOCARE Medical cat. EM897L).

#### Digital Imaging of IHC Slides

Upon completion of the staining the slides were scanned at 20x magnification using the Vectra 2 (PerkinElmer Life and Analytical Sciences, Boston, MA). The spectral image consists of λ-stacks captured with an acusto-optic tunable filter in the spectral λ range between 420 and 700 nm with 20 nm resolution. The system is equipped with an 8-bit monochromatic camera and a 20X objective, and each image plane in the λ-stack has 1392 × 1040 pixels. The exposure time was 4 ms, and a 1 × 1 pixel binning was used for image acquisition (Nuance acquisition software, Perkin-Elmer). A flat-field correction was applied to prepare the cube for digital unmixing of chromophore colors. The pixel size of the Vectra systems is 0.5 × 0.5 μm. To separate the chromogenic signals, the raw spectral image stacks were imported into the InForm™ 2.0 tissue image analysis software (Perkin-Elmer, Waltham MA). Using a spectral library generated from single stains of each chromogen color, the images were unmixed and individual chromophore images were separated by the InForm™ software. Image visualization was accomplished by a multi-layer tiff image. After the spectral unmixing, a mask was generated using Matlab R2016a (Mathworks, Natick, MA) which included labels of cancerous and benign areas of the tissue as well as empty parts of the tile. Gray scale images of individual chromophores were used for downstream analysis.

#### Cancer Cell and T Cell Masks in IHC Images

Binary masks of cancer cells and T-cells were generated from unmixed images of IHC slides. The mask of cancer cells was obtained through the machine learning module built into the InForm ver. 2.0 software. The module was trained by the operator to automatically delineate the cancer epithelium. Fifty small cancer areas were randomly selected from across all slides to train the algorithm. The trained module was applied to image tiles from IHC slides and yielded binary masks with cancer epithelium in the foreground (white pixels) and the remaining tissue components in the background (black pixels). The cancer cell mask was transferred via image registration to IF stained slides.

#### IF and IHC Image Co-registration

We implemented the affine image co-registration procedure (37) to transfer the tumor or T cell masks from the IHC to the corresponding IF images based on nuclear staining intensities of hematoxylin and DAPI. The unmixed hematoxylin mask from the IHC and the DAPI mask from the IF were used in the co-registration procedure and to determine the co-registration transformation matrix (37). The matrix contains a set of parameters that control the transfer and alignment of the images. After establishing the co-registration parameters, masks were automatically transferred for all tiles in a case. The co-registered area in each tile was used to generate tumor and T cell masks analyzed for densities of CD68+, CD163+, CD11b+, and CD11c+ pixels.

#### Thresholding IF Staining and Pixel Area Quantification

The gray scale images of the IF images were thresholded using Matlab software. Thresholds were visually adjusted using multiple images from each cancer type. After thresholding, a binary image was created for each channel and image tile and positive pixel were enumerated. Pixel numbers were exported together with the area from which they were obtained. Pixel groups with fewer than 9 pixels were excluded from the analysis.

#### Nuclear Segmentation and Cell Phenotyping

A nuclear segmentation algorithm was applied to hematoxylin or DAPI images to outline nuclei and to output a nuclear mask. The nuclear outline was expanded into a doughnut by a fixed length equal to 1/3 of the mean nuclear radius and positive pixels were counted within the doughnut. If the positive pixel density exceeded a predefined threshold, the cell was classified as positive. The process was repeated for all antibody channels (38, 39).

#### Comparing Cell-Based and Pixel/Area-Based Segmentation Approaches

The positive pixels were detected in the field of view (FOV) by fluorescence intensity thresholding using the same threshold value for pixel- and cell-based segmentation methods. The experiment was carried out using tiles from inflamed colon mucosa (*n* = 28) and correlations between pixels and nuclear counts were evaluated using the Pearson correlation coefficient.

#### Pixel Designations in MC&M Populations

We used the binary masks from the pixel-based segmentation approach to analyze macrophage populations. The segmentation of pixels was performed in Matlab and the segmented pixels were stratified into several masks. The MC&M-mask consists of the union of positive pixels from all antibodies, while the other masks originate from individual antibodies. Pixels in these antibody masks possess one or more colors. Pixels in the CD68-mask and CD163-mask are divided into single, double and triple positive pixels, which are counted separately. A small number of residual pixels that are positive for 4 or 5 antibodies is not further separated.

##### Single positive pixels

Single positive pixels are pixels colored exclusively only by one of the antibodies. They are counted after excluding double and higher order labeled pixels from individual antibody masks.

##### Double positive pixels

Double positive pixels are pixels positive for two antibodies. They are generated by the intersection of two masks. Labels include CD68+/CD163+, CD68+/CD11b+, CD68+/CD11c+, CD163+/CD11b+, CD163+/CD11c+, CD11b+/CD11c+. Double positive pixels may contain small subgroups of triple and quadruple positive pixels.

##### Triple positive pixels

Triple positive pixels are pixels positive for three or more antibodies. They are identified by the overlap of pixels of 3 masks and contain a small population of 4 and 5 color positive pixels.

#### Pie-Charts

Pie charts in **Figure 4A** consist of single positive CD68+, CD163+, CD11b+, CD11c+, and P2,3,4,5 pixel groups. For each pixel class, the average across all the tiles from a case is calculated and shown in the pie-chart. The related standard deviations are listed in Supplementary Tables.

Pie charts in **Figure 4B** illustrate in detail the double positive and higher order populations shown in **Figure 4A**. Double positive pixels are obtained directly from dichotomized gray-scale images using a Matlab code and by overlaying two individual color masks. Higher order pixel numbers are obtained by subtracting single and double positive pixels from the MC&M-mask.

Pie charts in **Figures 4C,D** illustrate single and multicolor pixel populations underneath CD68-masks or CD163-masks. Double positive pixel populations include CD68+/CD163+, CD68+/CD11b+, CD68+/CD11c+ and CD163+/CD11b+, CD163+/CD11c+. Triple positive pixel populations include CD68+/CD163+/CD206+, CD68+/CD11b+/CD11c+ and CD163+/CD11b+/CD11c+. All other triple positive and quadruple positive pixels exist at a frequency below 3.0% and are not included in the pie-charts.

#### Measuring Densities and Distances

Densities of pixels belonging to CD68, CD163, CD11b, and CD11c-masks were measured inside and outside the tumor mask and underneath the T cell mask. In mIF and mIHC co-registered images, the number of each pixel color was dived by the number of cytokeratin positive pixels (tumor area). MC&M pixel groups of fewer than 9 pixels were excluded from the analysis.

We measured two types of distances: between MC&M pixels and tumor cells, and between MC&M pixels and T cells. To measure the distances, we identified tumor cell nuclei located at the tumor periphery in mIF/mIHC co-registered images. These nuclei were identified by first overlaying the tumor mask on the nuclear mask from the IHC slides and then excluding all nuclei not located within a region demarcated at the edge of the tumor mask by an isometric line. The tumor border region was transferred to individual IF images to measure the distances between tumor nuclei and MC&M pixels.

To find the closest group of MC&M pixels to each tumor cell nucleus at the tumor border we wrote an algorithm that employs two-dimensional Euclidean distance transform and identifies shortest distance between the nucleus and the closest MC&M antibody pixel. For each tumor nucleus, four distances were returned by this algorithm, one for each MC&M antibody. The same algorithm was used to measure distances between T cells and MC&M pixel classes. Proximities between MC&M pixel classes and T cells, and between MC&M pixel classes and tumor cells were illustrated through distance histograms. The distances were binned by distance interval (0, 0–20 microns, 20–40 microns etc.).

#### PLEXODY Assay

This assay consists of the following pipeline: multiplex IF (mIF) staining→ digital image acquisition (fluorescent labels)→ multiplex IHC (mIHC) staining→ image acquisition (chromogenic labels)→ co-registration of mIF and mIHC tiles→ digital image analysis and data generation. The data are analyzed with in house software programs with Matlab.

#### Statistical Analysis and Data Visualization

Data were analyzed in Microsoft Excel using built in functions for *t*-test, Pearson correlation coefficient, and ANOVA. Graphs for data visualization were generated in Microsoft Excel.

## Results

### A Multiplex Assay for Myeloid Cells and Macrophages

**Figure 1:**
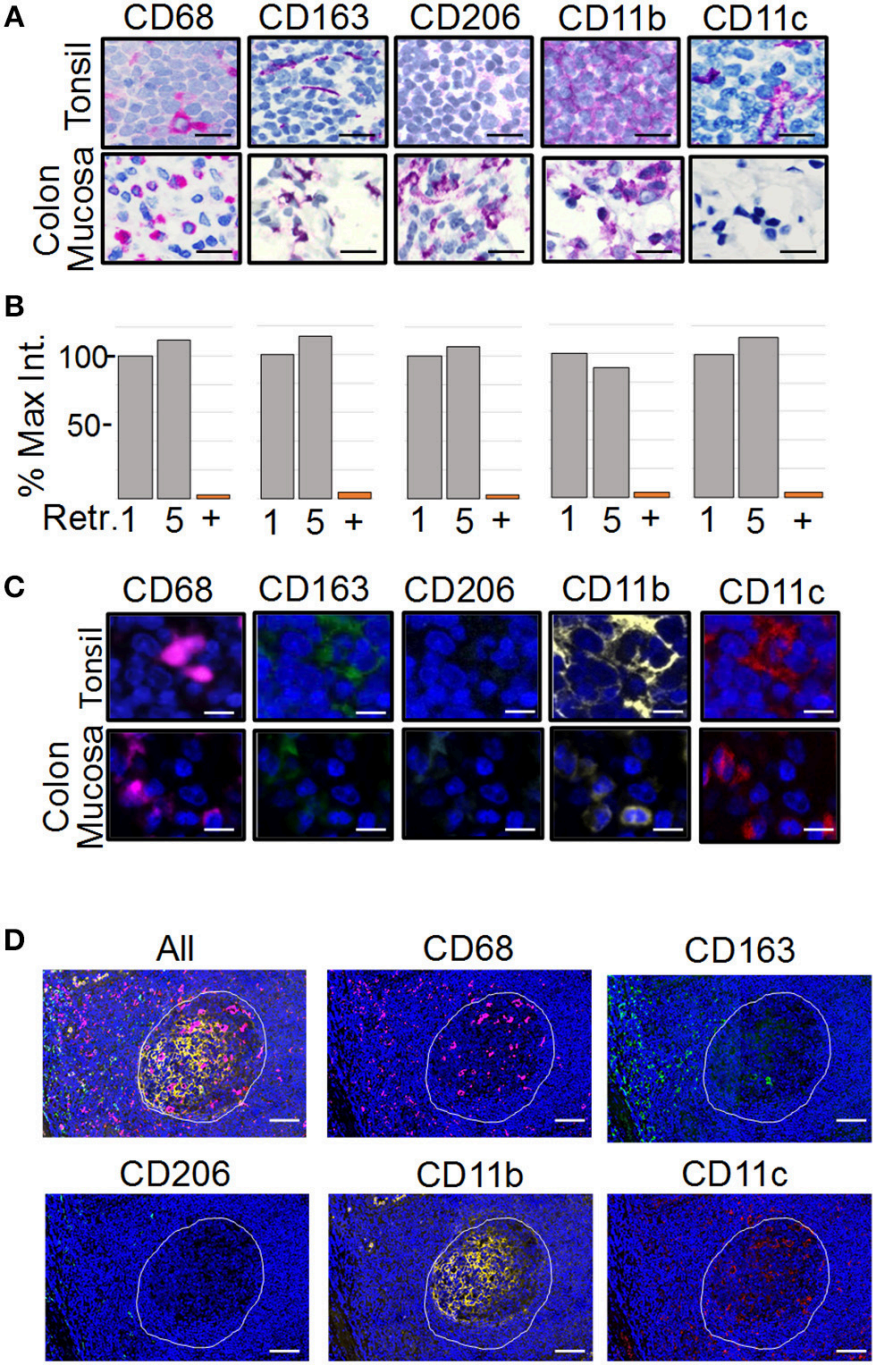
Development of the PLEXODY multiplex tissue staining assay. **(A)** *Antibody staining patterns using chromogenic IHC detection*. Panels depict select regions in tonsil (upper row) or inflamed colon mucosa (lower row) stained with the antibody listed above each panel. Slides were counterstained using hematoxylin. Scale bar = 20 μm. **(B)** *Sensitivity of antibodies to antigen retrieval and heat denaturation*. Antibody binding was measured by chromogenic signal intensity in tissues subjected to one or five rounds of antigen retrieval treatments. The signal intensity in the histogram after one retrieval was considered 100%. *P*-values are shown in Supplementary Table 1. In addition, each antibody was removed by heat denaturation and the slide was tested for residual antibody binding using the secondary antibody with a red chromogen. The percentage of signal remaining after antibody removal is shown as a bar labeled (+). **(C)** *Antibody staining patterns using mIF*. Panels depict a region in tonsil or inflamed colon mucosa. Slides were counterstained using DAPI. Scale bar = 10 μm. **(D)** *Antibody staining patterns in tonsil*. The region of tonsil includes a germinal center (white outline) and perifollicular zone surrounding the germinal center. Scale bar = 200 μm.

To analyze the spatial organization of tumor-associated myeloid cells and macrophages (MC&M) through visualization by antibodies, we developed an automated, multiplexed immunofluorescent assay. We optimized the staining conditions for anti-CD68, -CD163, -CD206, -CD11b, and -CD11c antibodies using immunohistochemistry (IHC) and the Ventana purple substrate, which revealed different staining patterns of each antibody in tonsilar germinal centers and inflamed colon mucosa (Figure 1A). The antibody clones used for the staining are either commonly applied in clinical practice (CD68 and CD163 prediluted Ventana formulations of validated monoclonal antibodies (40, 41), have been validated by the vendor (Abcam rabbit monoclonal antibodies CD11b, CD11c), or have been tested in The Human Protein Atlas (www.proteinatlas.org). CD68+, CD163+, CD11b+, and CD11c+ cells where observed in tonsil and colon. In contrast, no CD206+ cells were present in the tonsil. However, CD206+ macrophages were noted in inflamed colon mucosa (Figure 1A). The non-overlapping staining patterns of MC&M-related antibodies in tonsil and colon confirm the specificities of the antibodies and assay conditions.

The multiplex assay consists of sequential staining rounds on the Ventana autostainer. Each round requires that the primary and secondary antibodies from the previous round are completely removed. This is accomplished by heat retrieval using citrate buffer pH 6.0. The position of the antibody in the multiplex panel, whether it is the first, second, third, fourth or fifth antibody is chosen in part, based on the sensitivity of the epitope to heat retrieval. Therefore, we tested the sensitivity to heat retrieval for each antibody. As shown in Figure 1B, none of the antibodies was affected by five rounds of heat retrieval, simplifying the design of the multiplex panel. Next, we determined the efficiency of removing antibodies after the staining cycle, which is another factor that determines the antibody sequence in the multiplex assay. Staining intensities before and after removal of each antibody by heat retrieval were compared. All antibodies were removed, excluding the possibility that overlapping signals are caused by residual antibody binding after heat retrieval (Figure 1B, column labeled with “+”).

As a next step, the chromogenic IHC assay was transferred to an immunofluorescent (IF) readout and multiplexed (Figure 1C). Since the amplification of the IHC and IF Ventana systems are comparable, antibody dilutions and incubation times remained the same. Individual staining patterns of antibodies in the IF 5-plex assay mimicked the single antibody IHC staining patterns in Figure 1A. In addition, changing the sequence of the antibodies in the panel did not alter staining patterns, suggesting that the tissue-bound fluorophores are not causing steric hindrance. Fluorescent slides were scanned on the TissueFaxs slide scanner using individual filter cubes that were harmonized with the emission wavelengths of the Ventana fluorophores. The imaging using filter cubes is not compromised by the bleed over of fluorophore signals and can be used with fluorescent signals of different staining intensities.

As tonsil is commonly used for validation of mIF assays (28), we compared the staining patterns of the MC&M reactive antibodies in germinal center and the perifollicular zone (Figure 1D). CD68+ pixels were abundant in germinal centers, while CD163+ pixels primarily populated the perifollicular zone. Both CD11b+ pixels and CD11c+ pixels resided inside germinal centers and to a lesser extent in the perifollicular zone. Overall, staining patterns and intensities during assay optimization and repeated testing of the 5-plex IF antibody panel were consistent between days.

### A Pixel-Based Detection Approach to Quantify Myeloid Cells and Macrophages

**Figure 2:**
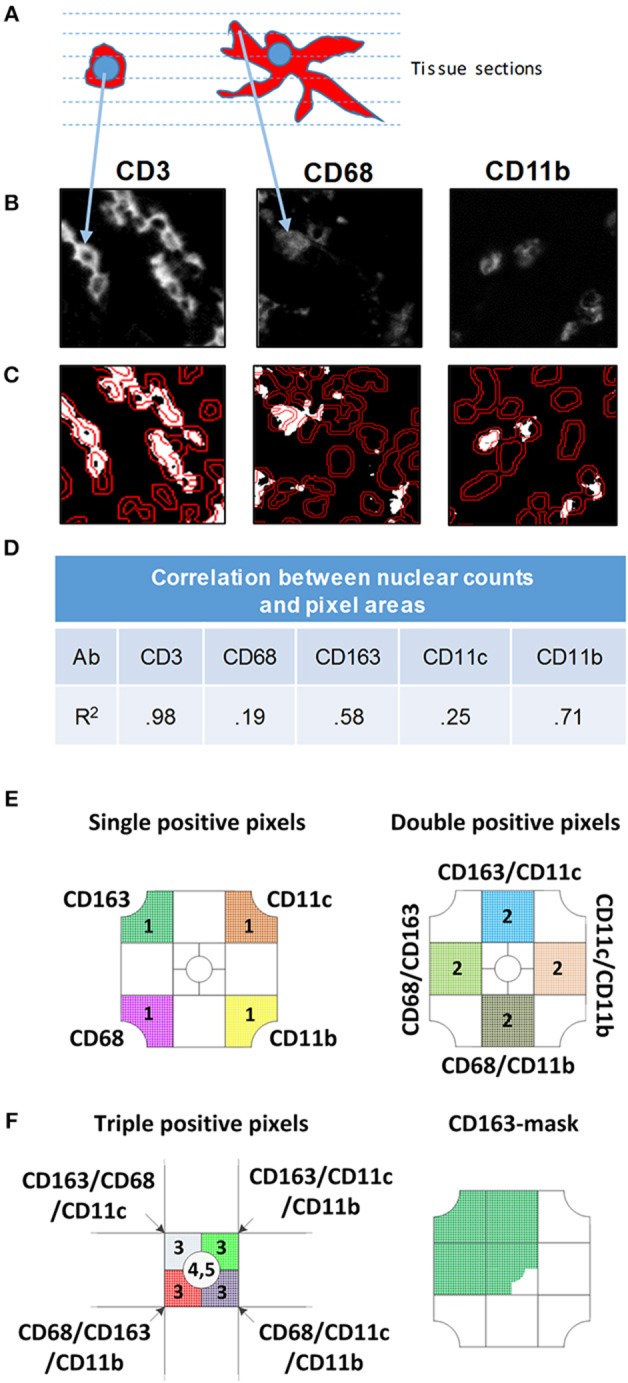
Comparison of nuclear and pixel-based segmentation methods. **(A)** Schematic image of a lymphocyte (left) and macrophage (right). The dashed lines represent virtual tissue sections. **(B)** Gray scale images of antibodies indicated above each panel. **(C)** Nuclear segmentation. Nuclei are outlined in the DAPI DAPI channel. The inner line demarcates the nucleus and the outer line shows the border of the doughnut used to quantify pixels for nuclear classification. To count positive pixels, the gray scale image was converted to a binary mask and thresholded. **(D)** Correlation between nuclear and pixel/area-based measurements. MC&M counts obtained through nuclear segmentation were correlated with MC&M-associated pixel areas obtained by the pixel-based segmentation. Pearson correlation coefficients (*R*^2^) are shown in the table. **(E)** *Schematic representation of single, double and triple positive pixel classes*. The single positive pixel group reacts with only one of the antibodies and consists of pixels stained with only on color. Pixels in double positive pixel groups, of which 10 classes can be identified, contain two colors. These pixels are identified by intersecting pixel masks from two antibodies and extracting those pixels with 2 colors. Triple positive pixels are obtained by the same process through intersection of binary pixel masks from three antibodies. **(F)** *Pixel mask of one antibody*. Example showing the CD163-mask. Pixels in this mask can be one, two, three, or more colors.

A previous study demonstrated that the nuclear-based image segmentation method may not accurately report macrophage numbers after staining with CD68 and CD163 because cellular processes can reach across long distances from nucleus (42). Fluorescently labeled cell processes are often in a different tissue section than the nucleus and an approach involving only perinuclear analysis will not lead to an acute measurement of macrophages (Figure 2A). Instead, a pixel-based segmentation approach, which includes the antibody signal in cellular processes is better suited for quantification of macrophages. Therefore, we used the pixel-based segmentation approach to identify positive pixels after setting a threshold (Figure 2B). Thresholds were based on the negative isotype controls included in every staining batch (not shown) and to remove artifacts, groups of fewer than 9 adjacent pixels were excluded from further analysis. Pixels were converted into a binary positive/negative mask for analysis (Figure 2C). The area encompassed by positive pixels was determined. For nuclear based segmentation and classification as positive or negative, we outlined nuclei in the DAPI channel and determined the average staining intensity normalized to area within a doughnut around the nucleus (Figure 2C). To determine how pixel-based and nuclear segmentation-results correlated across antibodies, we used 28 tiles of inflamed colon mucosa to calculate correlation coefficients between nuclear counts (cell number) and pixel counts (cell area) (Figure 2D, Supplementary Figure 1). Correlation coefficients differed for each antibody. The lowest was for CD68 (*r* = 0.19) and CD11c (*r* = 0.25), while CD11b revealed good concordance between nuclear and pixel-based quantification methods (*r* = 0.71). As a control, we measured the correlation for CD3+ T cells, since cellular processes are rare in T cells and all positive pixels are in the perinuclear region. As expected, the correlation between the cell and area measurements was high (*r* = 0.98). Thus, we confirmed a previous study demonstrating that the pixel-based analysis approach is better suited for quantification of MC&M-associated pixels than the nuclear segmentation-based method (42) and proceeded to use the pixel-based segmentation method to analyze the spatial organization of MC&M-associated antibody staining patterns.

The pixel-based segmentation can be used to count pixels that are colored with one, two, three or more antibodies by overlaying and intersecting individual pixel masks (Figure 2E). We measured single positive pixel classes (*n* = 4) only containing one color. Double positive pixel classes (*n* = 10), are generated by the intersection of pixels from two pixel masks and triple positive pixel classes are obtained by the intersection of three masks. The CD206 mask is included. Alternatively, a pixel mask is generated for only one antibody, for example for CD163, and pixel colors underneath the mask are analyzed (Figure 2E). There will be single, double, triple and higher order colored pixels within this CD163-mask, which can be further quantified. Altogether, the pixel-based segmentation approach can be used to measure the areas that are occupied by various pixel classes and to infer spatial locations of MC&M cells that are marked by these pixels. However, the pixel-based segmentation approach cannot be used to enumerate MC&M subpopulations, because to the most part, pixels cannot be assigned to specific cells and it is unknown whether individual pixel classes are uniform and consistent within cellular subgroups MC&Ms.

**Figure 3:**
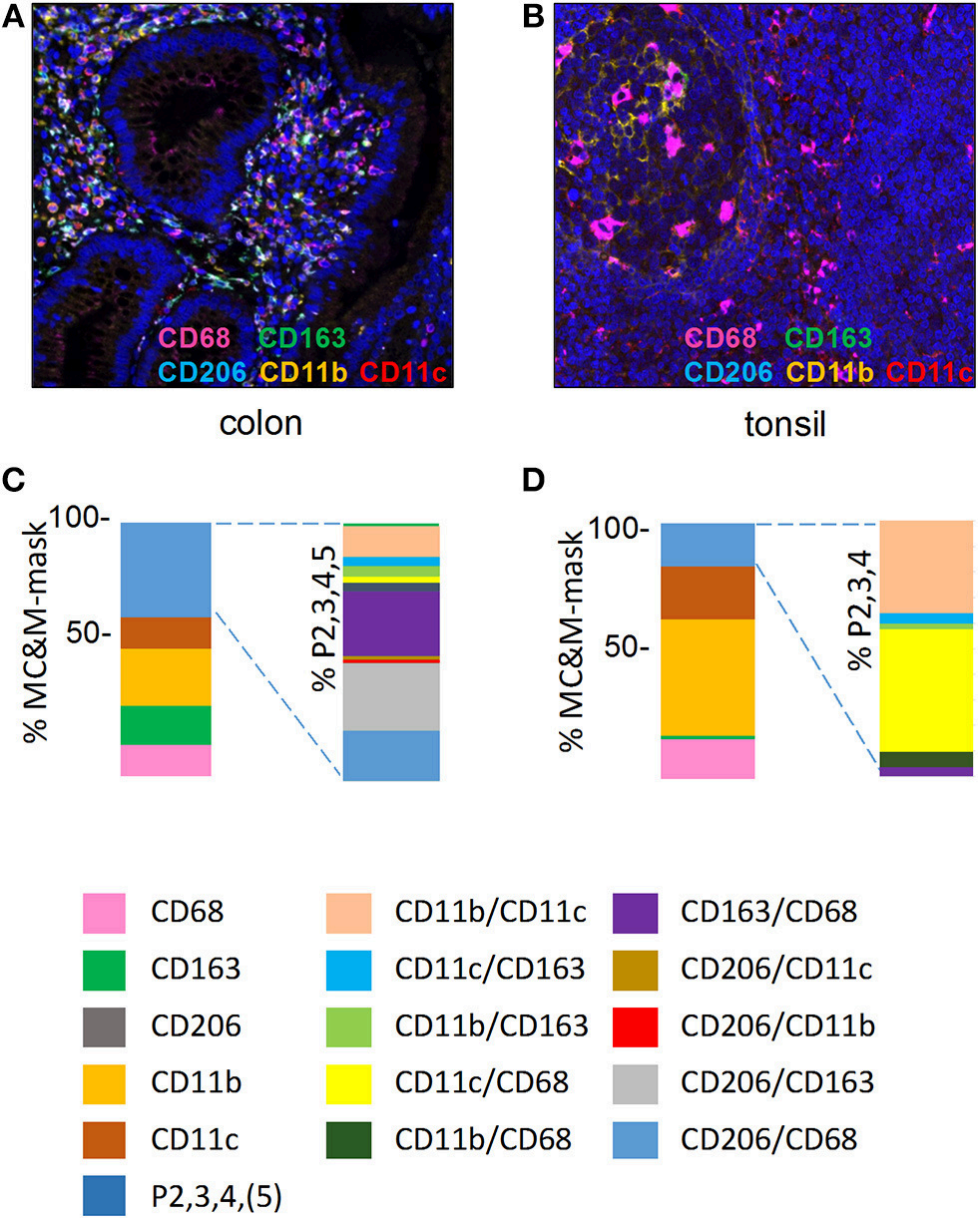
MC&M-associated pixels in inflamed colon mucosa and tonsillar germinal centers. **(A**,**C)** *Colon mucosa*. **(A)** Representative image tile of mIF 5-plex. *C. Frequencies of single and double positive MC&M-associated pixel classes*. The MC&M-mask, determined by the intersection of CD68+, CD163+, CD206+, CD11b+, and CD11c+ pixels, was used as the reference. Single positive and multicolor (P2, 3, 4, 5) pixel numbers were extracted from 28 tiles and the percentages of MC&M mask are shown in the left stacked bar. Standard deviations are in Supplementary Table 2. The P2, 3, 4, 5 pixel classes are further separated into ten two-color MC&M classes and their percentages are shown in the right stacked bar. **(B,D)** *Germinal center of tonsil*. **(B)** Representative image tile of mIF 5-plex. **(D)** *Frequencies of single and double positive MC&M-associated pixel classes*. The MC&M-mask, determined by the intersection of CD68+, CD163+, CD11b+, and CD11c+ pixels, was used as the reference. CD206+ pixels were not observed. Single positive and multicolor, P2, 3, 4 pixel numbers were extracted from eight tiles and their percentages of the MC&M-mask are shown in the left stacked bar. Standard deviations are in Supplementary Table 3. The P2, 3, 4 pixels were further separated into six two-color MC&M-associated pixel classes and their percentages are shown in the right stacked bar.

Next, MC&M populations visualized by the 5-plex assay were quantified in inflamed colon mucosa and germinal center of tonsil (Figures 3A,B, Supplementary Figures 2,3). We measured pixels colored by one or two antibodies and generated 14 separate classes of MC&M-associated pixels. To calculate the percentage of single and double color pixels, we used the union of MC&M-associated pixels (MC&M-mask) as the reference, which is the area in the tissue taken up by all positive pixels. Eight pixel classes dominated in colon mucosa compared to six in tonsil germinal center. Further, 55% of pixels in the colon mucosa were reactive with only one antibody (single positive pixels) while in the germinal centers 84% of pixels were single positive (Figures 3C,D). In the colon mucosa, the most abundant population of single positive pixels was CD11b+ (20%), while CD68+, CD163+, and CD11c+ pixels each encompassed approximately 10% of the MC&M mask, which is the area defined by the intersection of the CD68, CD163, CD11b, and CD11c colored pixels. In the germinal centers of the tonsil, CD11b+ single positive pixels amounted to 45% of the MC&M-mask and the percentage of double positive, P2 pixels was reduced compared to colon mucosa (Figure 3D).

In inflamed colon, we included CD206+ pixels in the double positive pixel category. As we observed CD206+ staining in vascular cells, we only measured CD206+ pixels underneath the MC&M-mask. The largest pixel fraction was CD163+/CD206+ (Figure 3C, Supplementary Table 2). In contrast to the colon mucosa, which displayed 10 double positive pixel classes, only 6 double positive pixel classes were observed in the tonsil germinal center (Figure 3D, Supplementary Table 3). The majority (90%) were CD68+/CD11c+ and CD11b+/CD11c+. Interestingly, despite the large number of CD11b+ pixels, CD68+/CD11b+ pixels were infrequent, suggesting that amount to which pixels overlap is not related to the abundance of pixels. This observation suggests that the overlap of pixels is specific and that the size of the pixels is small enough to avoid overlap due to crowding. Together these results demonstrate the organ specific differences of MC&M-associated pixel classes.

### The Combined mIF and mIHC Plexody Assay

As a next step, we built a prototype assay that consisted of the 5-plex mIF, followed by a 3-plex mIHC assay. This assay, which we named PLEXODY, was used to map the spatial characteristics of MC&M populations in tumor areas and their relationships to tumor and T cells. As a proof-of-principle application we stained three cases each of pancreatic, prostate and kidney cancer. After automated staining of one slide per case, the tissue was scanned on the TissueFaxs. Digital IHC images were acquired on the Vectra 2.0 multispectral imaging system and colors were unmixed using the InForm™ software. The tiles from TissueFaxs and Vectra were co-registered based on nuclear outlines to generate the dataset for analysis.

**Figure 4:**
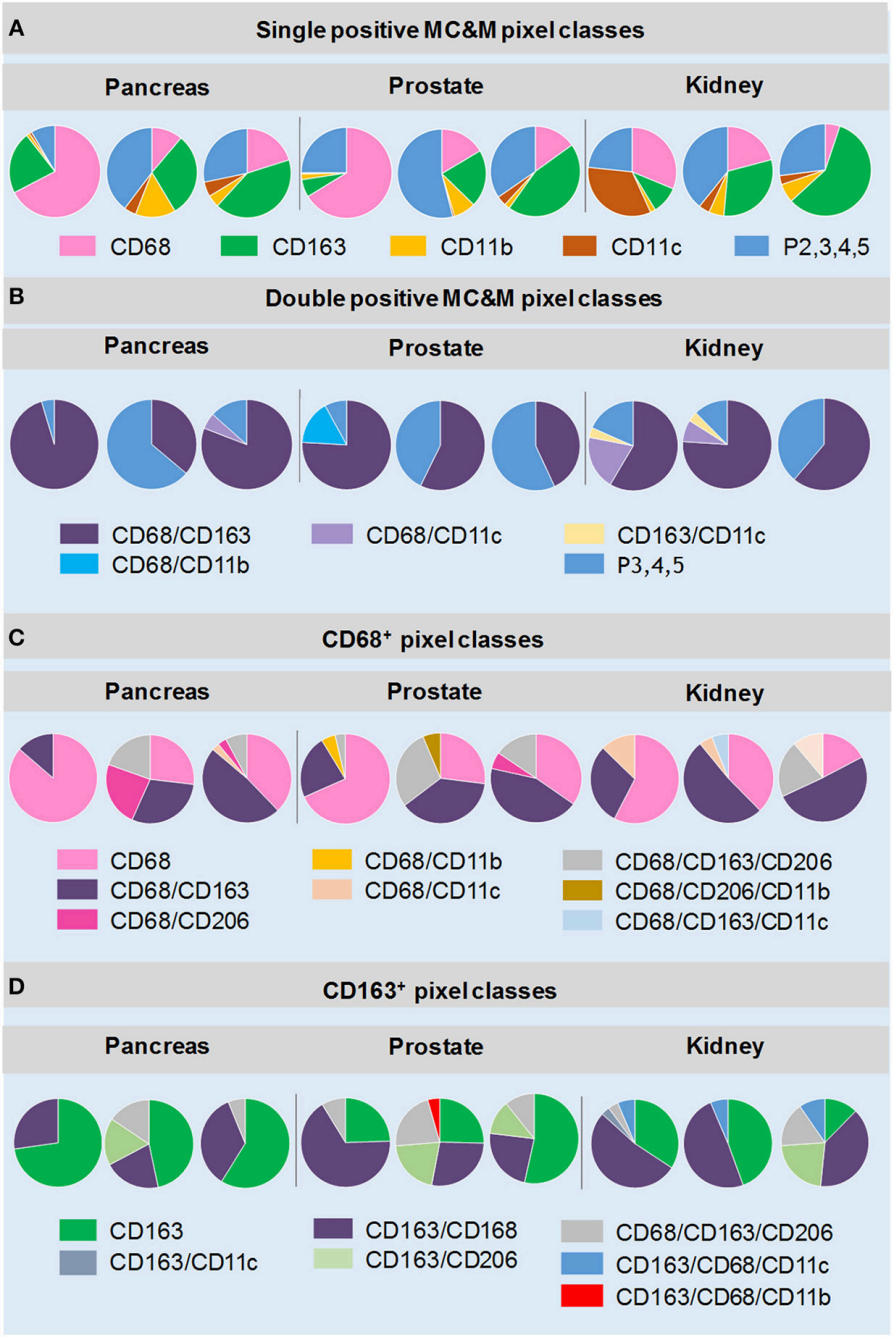
MC&M-associated pixel classes in cancers from pancreas, prostate and kidney. MC&M-associated pixel classes at a frequency of >3% are shown for three cancer types (pancreatic ductal adenocarcinoma, prostate adenocarcinoma, and clear cell renal cell carcinoma). **(A)** *Single positive MC&M-associated pixel classes*. Pie charts show single color CD68+, CD163+, CD11b+ and CD11c+ pixels as a fraction of the MC&M-mask for each cancer case. The P2,3,4,5 fraction includes all pixels colored by more than one antibody. **(B)** *Double positive MC&M-associated pixel classes*. Pie charts show double positive pixel classes within the P2, 3, 4, 5 pixel group for each cancer. In addition, the P3, 4, 5 fraction, which are pixels colored by more than two antibodies are shown. **(C)** *Pixel classes within the CD68-mask*. Single, double and triple positive pixels are shown as a fraction of all CD68+ pixels (CD68-mask). **(D)** *Pixel classes within the CD163-mask*. Single, double and triple positive pixels are shown as a fraction of the CD163+ pixels (CD163-mask). Values in pie-charts, standard deviations and number of tiles analyzed per case are in Supplementary Tables 4–7.

The analysis first focused on tissue areas positive for CD68, CD163, CD11b and CD11c. Again, CD206 staining, which appeared in MC&Ms and endothelial cells of tumor vasculature (Supplementary Figures 4–12) was only analyzed underneath the MC&M-mask. Figure 4 shows the results of 9 cancer cases, illustrated in Supplementary Figures 4–12. Across cancer types, percentages of single positive CD68+ and CD163+ pixels, ranged from 6.5 to 62.7%, while CD11b+ and CD11c+ single pixels ranged from 0.65 to 11%. Percentages of double and higher level positive pixels (P2,3,4,5) comprised 9.7–56% of the MC&M-mask. (Figure 4A, Supplementary Table 4). Next, we further dissected the P2,3,4,5+ pixel populations by quantifying four double positive pixel classes, CD68+/CD163+, CD68+/CD11b+, CD68+/CD11c+, and CD163+/CD11c+. The CD68+/CD163+ class was most abundant, comprising 40–92% of all P2 pixels (Figure 4B, Supplementary Table 5). The higher order colored pixels stained with 3–5 antibodies occupied 3.8–21.8% of the P2 positive pixel area. From these data, we conclude that the majority of the P2,3,4,5 pixel group are positive for 2 colors.

Next, we quantified pixel classes underneath the CD68+ and CD163+ pixel masks as illustrated in Figure 2E (Figures 4C,D, Supplementary Tables 6, 7). Approximately 23–85% of pixels underneath the CD68-mask were single color. The most abundant double positive pixel class was CD68+/CD163+ and the most abundant triple positive pixel class was CD68+/CD163+/CD206+ (up to 29% of CD68-mask in a case of prostate cancer, Supplementary Table 6). Interestingly, a unique feature in renal cancer was an abundance of CD68+/CD11c+ (2.2–13% of the CD68-mask) and CD68+/CD163+/CD11c+ (3.5–15%) pixels. We also analyzed pixels underneath the CD163-mask. Similar to CD68+ pixels, the majority of CD163+ pixels were single positive (6–72% of CD163-masks). However, in some cases, double positive CD163+/CD68+ pixel frequencies exceed those of single positive pixels. As expected high frequencies of CD163+/CD206+ and CD163+/CD206+/CD68+ pixels were observed. Interestingly we observed two cancer groups with regards to the frequencies of CD163+/CD206+ pixels, independent of cancer type. The high CD163+/CD206+ cancer group consisted of 4 cases (one pancreas, two prostate, one kidney cancers) with 11.5–26.3% of the CD163 mask comprised of CD163+/CD206+ pixels. The low CD163+/CD206+ included the other 5 of the 9 cancer cases with frequencies of CD163/CD206+ pixels less than 3% of the CD163-mask. In summary, the analysis of MC&M-associated pixel classes revealed a large amount of heterogeneity within and across cancer types with no clear patterns that are cancer type specific.

**Figure 5:**
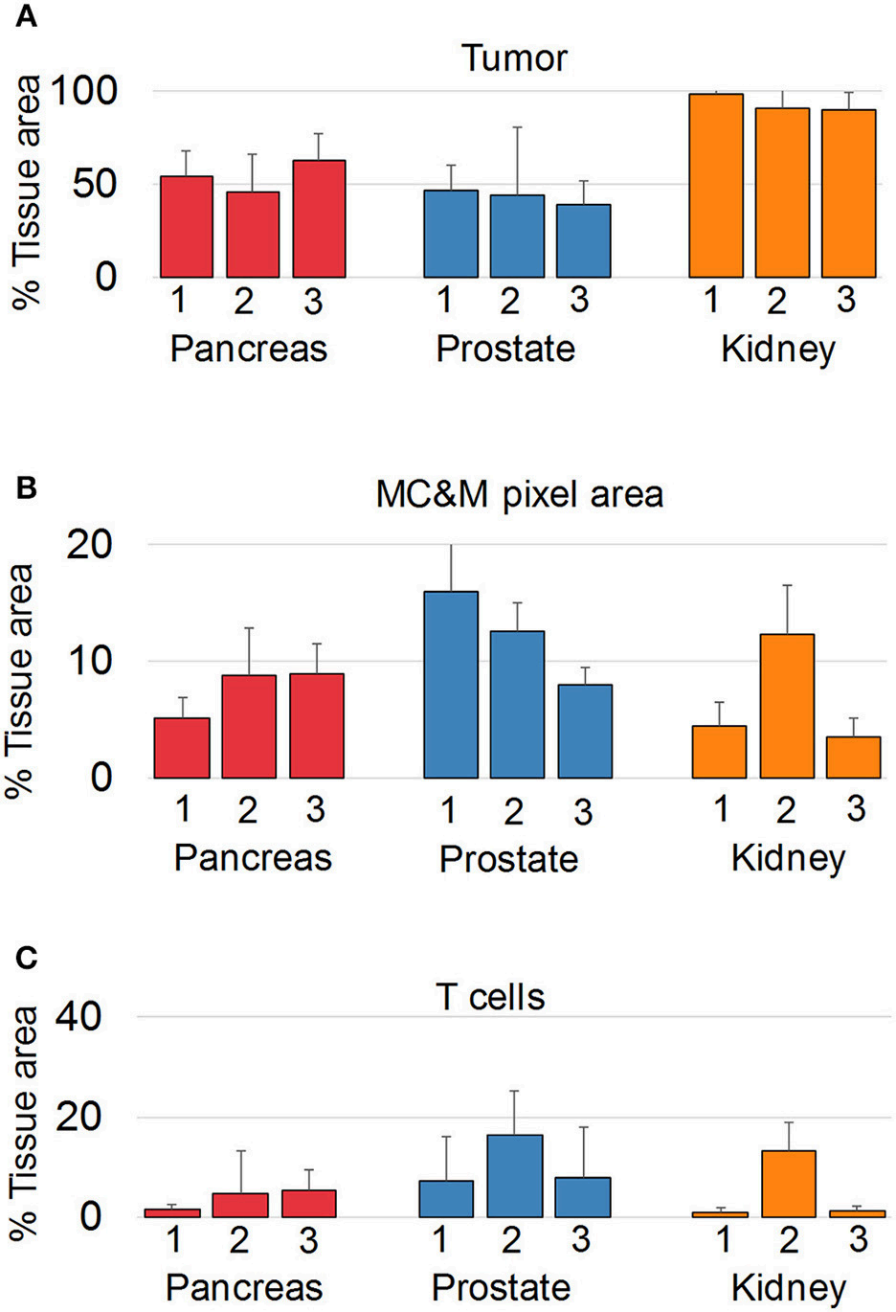
Multiplex antibody staining with sequential IF and IHC (PLEXODY assay). Fluorescent and chromogenic masks from each tile are integrated into a single dataset. **(A)** *Percentage of tissue area occupied by cancer*, and positive for cytokeratins 8/18. Standard deviations are indicated by the line above each bar. In prostate cancer cases, tumor and normal glands were separated based on mIHC with E34β12, an antibody binding to high-molecular weight cytokeratins in basal cell of normal glands. Corresponding representative digital images of mIF and mIHC in Supplementary Figures 4–12. Values and tile numbers in Supplementary Table 8. **(B)** *Percentage of tissue area encompassed by MC&M-mask stained by mIF*. **(C)** *Percentage of tissue area positive for CD3*.

Since we did not observe cancer specific patterns of MC&M-associated pixel classes, we questioned whether pixels differ in spatial organization. We employed the PLEXODY assay to generate tissues stained with the five MC&M-related antibodies, and in addition with antibodies against high and low molecular weight cytokeratin and CD3. To prepare image tiles for analysis, IF and IHC tiles were co-registered based on overlapping DAPI and hematoxylin nuclear intensities [Supplementary Figure 13, (39)]. In these tiles, the tumor area comprised 38.6–98.1% of the tissue (Figure 5, Supplementary Table 8), while the tissue area occupied by MC&M and T cell associated pixels amounted up to 16.0 and 16.5%, respectively.

**Figure 6:**
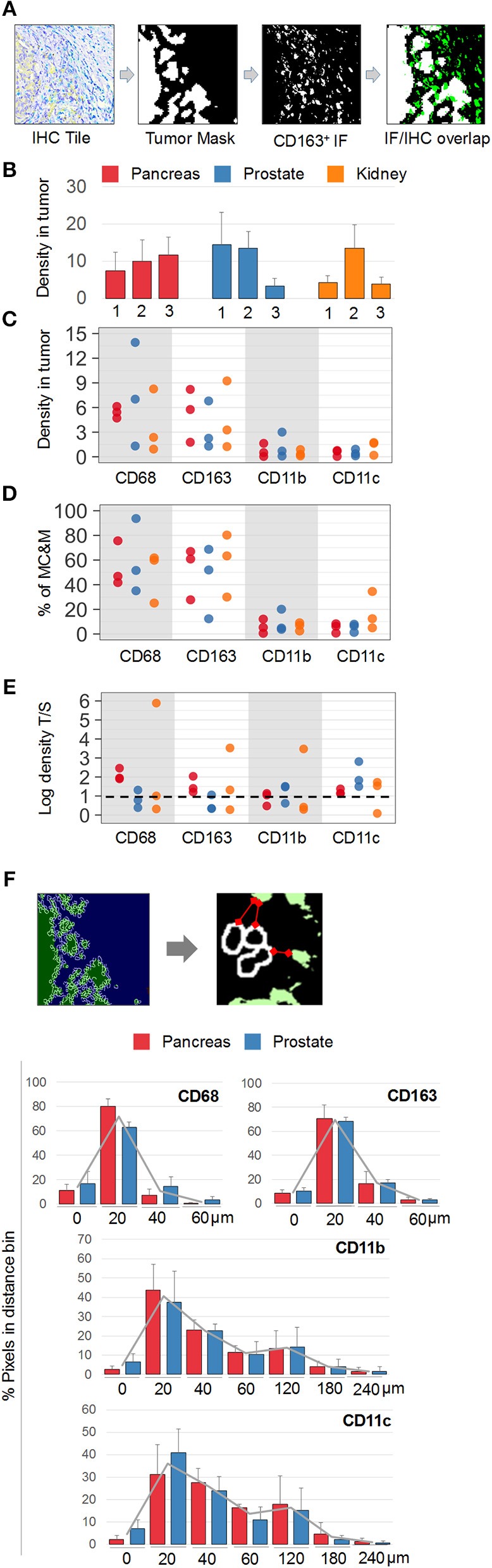
Spatial mapping of MC&M-associated pixel classes. **(A)** *Workflow diagram* of overlay between chromogenic cancer mask and fluorescent CD163-mask. **(B)** *Density of MC&M mask in tumor area*. For each cancer case, labeled 1, 2, or 3, the MC&M-associated pixel density underneath the tumor mask is shown by the bar. *N* = 6–10 tiles/case. Standard deviations are shown in Supplementary Table 9. **(C)** *Densities of MC&M-associated pixel classes in tumor area*. For each MC&M-associated pixel class, positive pixels underneath the tumor mask are divided by the area of the tumor mask. *N* = 6–10 tiles/case. Standard deviations are shown in Supplementary Table 9. **(D)** *Percentage of MC&M-associated pixel classes underneath the tumor mask*. The percentage of CD68+, CD163+, CD11b+, and CD11c+ pixels of the MC&M-mask in tumor areas is shown (*n* = 6–10 tiles per cancer type). Positive pixels include single, double, triple and higher order positive pixels. Standard deviations are shown in Supplementary Table 10. **(E)** *Tumor: stroma densities of MC&M-associated pixel classes*. Ratio of MC&M-associated pixel densities in the tumor **(B)** and MC&M-associated pixel density in the stroma. Stromal pixels are calculated by subtracting pixels in tumor area from total pixels in tissue. Standard deviations are shown in Supplementary Table 11. The dashed line refers to an equal density of colored pixels in the tumor and stroma **(F)**. *Distance measurements*. Schematic representation of distance measurements between nuclei at the tumor border and CD163+ pixel clusters. The segmentation of tumor nuclei is shown in the left panels. A representative measurement of the distance between a nucleus and the closest MC&M-associated pixel group is shown in the right panel. Histograms of tumor–MC&M-associated pixel distances in prostate (*n* = 25 tiles) and pancreas (*n* = 23 tiles) cancers. The average percentages of tumor–MC&M-associated pixel distances (y-axis) in a distance interval (x-axis) are shown with the standard deviations. Separate values for prostate and pancreatic cancer types are in Supplementary Table 12.

Next, MC&M-associated pixel densities were determined inside and outside the tumor (Figure 6A). Inside the tumor, densities of MC&M-associated pixels ranged from 3.4 to 14.5% (Figure 6B, Supplementary Table 9). Amongst pixel classes, CD68+ and CD163+ pixels predominated over CD11b+ and CD11c+ pixels (Figure 6B, Supplementary Table 10). CD68+ comprised between 25 and 94% of pixels within the MC&M mask inside the tumor (Figures 6C,D, Supplementary Table 10). Interestingly, the correlations between CD163+ pixels and either CD11b+ or CD11c+ pixels inside the tumor were *r* = 0.51 and *r* = 0.44, respectively, while other pairwise correlations were less than *r* = 0.2. Finally, we compared densities of MC&M-associated pixel classes in tumor and stromal regions (Figure 6E, Supplementary Table 11). In 11/27 cases, densities were greater in the tumor region compared to the stroma. For each antibody, there were more cases with a greater density of MC&M-associated pixels inside the tumor compared to the stroma, suggesting that MC&Ms are recruited into tumor areas.

In addition to local densities of MC&M-associated pixels, we measured their distances from the edge of the tumor (Figure 6F, Supplementary Table 12). Interestingly, distances varied by pixel class, but not by cancer type. Data were generated for prostate and pancreas, but not for renal cancer because of the tumor growth pattern. An average 70% of CD68+ and CD163+ pixels were between 0 and 20 microns from the tumor edge. In contrast, only 40% of CD11b+ pixels and 36% of CD11c+ pixels were within 0 and 20 microns of the tumor boundary, with most pixels at a greater distance from the tumor. CD11b+ and CD11c+ pixels were identified at a 240-micron distance, while CD68+ and CD163+ pixels did not exist more than 180 microns away from the tumor. In summary, densities of CD68+ and CD163+ pixels are greater within and close to the edge of the tumor, consistent with attraction of macrophages by the tumor while CD11b+ and CD11c+ pixels reside at a greater distance from the tumor.

**Figure 7:**
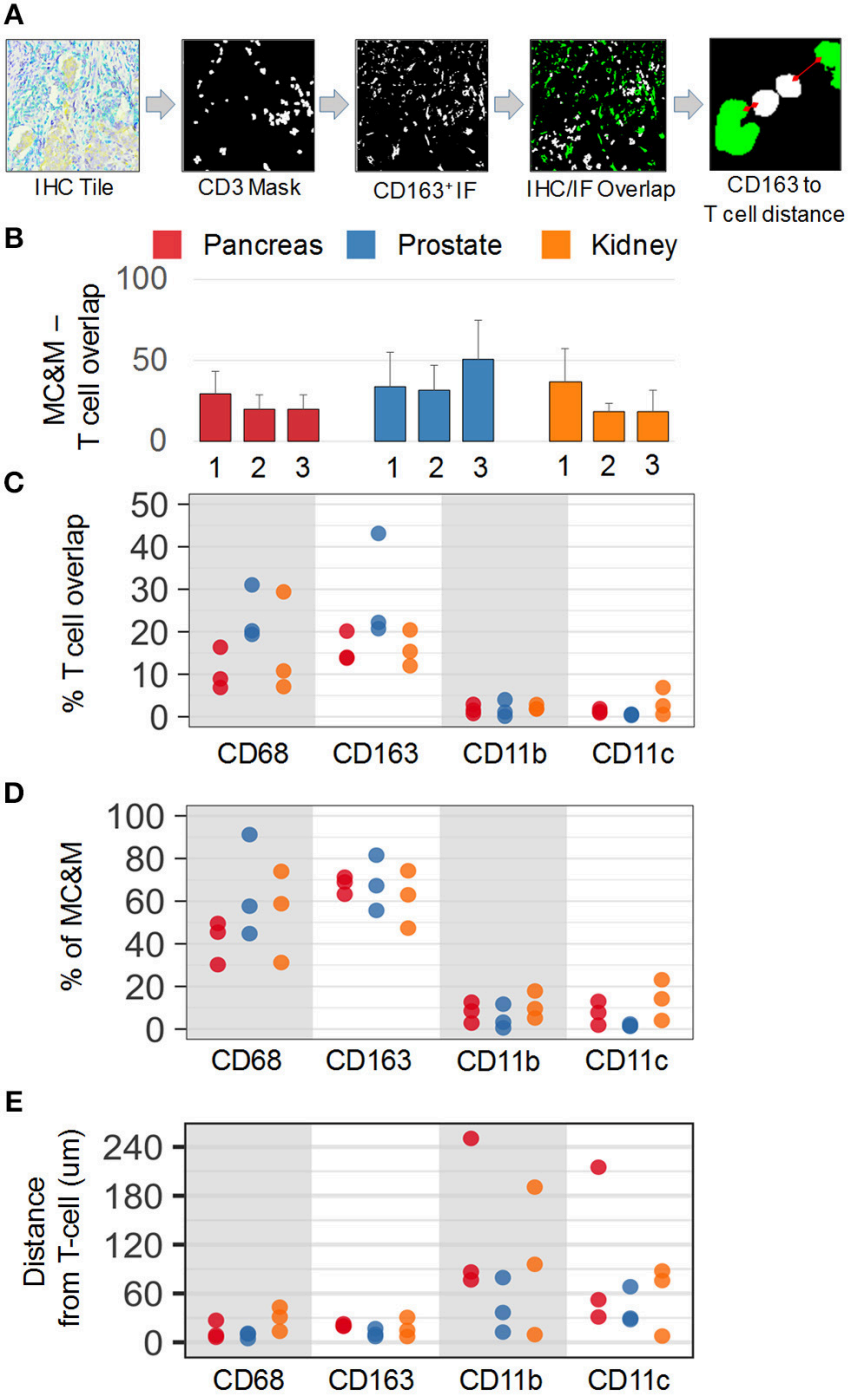
Spatial relationships of MC&M-associated pixel classes and CD3+ T cells**. (A)** *Workflow diagram* of overlay of CD3+ T cell mask and CD163-mask in tile labeled IHC/IF and illustration of distance measurement. **(B)** *Densities of antibody masks in direct contact with T cells*. For each cancer case, labeled 1, 2, or 3, the MC&M-associated pixel density underneath the CD3-mask is shown by the bar. *N* = 6–10 tiles/case. Standard deviations are shown in Supplementary Table 13. **(C)** *Densities of MC&M-associated pixel classes in direct contact with T cells*. For each MC&M-associated pixel class, positive pixels underneath the CD3 mask are divided by the area of the CD3 mask. *N* = 6–10 tiles/case. Standard deviations are shown in Supplementary Table 13. **(D)** *Percentage of MC&M-associated pixel classes underneath CD3 mask*. The percentage of CD68+, CD163+, CD11b+, and CD11c+ pixels of the MC&M-mask in contact with T cells is shown (*n* = 6–10 tiles per cancer type). For each antibody, positive pixels include single, double, triple and quadruple positive pixels. Standard deviations are shown in Supplementary Table 14. **(E)** *Mean distances between T cells and MC&M-associated pixel classes*. Distances were measured as shown in **(A)**. The mean distance and standard deviation are shown for each case and antibody in Supplementary Table 15.

In addition to analyzing the spatial relationship of MC&M-associated pixel populations and tumor cells, we performed a similar analysis for pixel classes and CD3+ T cells. In co-registered IF and IHC tiles, we identified the T cell mask based on positive staining for CD3 (Figure 7A). Compared to tumor areas with a maximal density of 14.5% of MC&M-associated pixels, MC&M-associated pixel densities underneath the T cell mask ranged between 18.5 and 51% (Figure 7B, Supplementary Table 13). Similar to the cancer regions, most MC&M-associated pixels in contact with T cells were CD68+ and CD163+. The average percentage of CD68+ and CD163+ pixels overlapping with CD3+ pixels was 53.6 and 58.3%, respectively (Figure 7C, Supplementary Table 13). In contrast, the percentage of CD3+ and CD11b+ or CD11c+ overlapping pixels averaged only 8 and 7.7%, respectively (Figure 7D, Supplementary Table 14). Mean distances from the T cell border amounted to 21.2 microns and 28.6 microns, respectively, for CD68+ and CD163+ pixels, while CD11b+ and CD11c+ pixels were on average 77.5 microns and 59 microns away from the CD3 mask (Figure 7E, Supplementary Table 15). Altogether, the data demonstrate higher densities and closer proximities of CD68+ and CD163+ pixels in tumor and T cell regions compared to CD11b+ and CD11c+ pixels.

## Discussion

We established a multiplexed fluorescent and chromogenic tissue staining assay, named PLEXODY, in which we apply up to 8 antibodies on tissue sections using a fully automated staining process. The mIF panels requires a 14-h staining program and the mIHC, an 8-h staining cycle. After co-registration of digital images from slides stained sequentially with CD68, CD163, CD206, CD11b and CD11c by mIF and with high molecular weight CK (clone 34E1β2), CK8/18 and CD3 by mIHC, we generated intersecting masks of up to 3 pixel colors and measured single, double and triple positive pixels defining 25 MC&M-associated pixel classes. While the IF component provides opportunities for visualization of multi-color MC&M-associated pixel classes, the IHC component adds diagnostic accuracy. Since commercial software applications are based on nuclear segmentation for quantification of multicolor digital slides, the stellate morphology and dendritic nature of some MC&M subtypes hinder the nuclear quantification approach. Therefore, we pursued an area-based quantification that does not rely on nuclear segmentation (Figure 2). Spatial distribution of MC&M-associated pixels differed between two normal tissue sources and between normal and cancer. In addition, the data provide novel insights into tumor associated MC&M populations: (i) the majority of MC&M-associated pixels are colored with only one antibody; (ii) CD68+/CD163+ predominate amongst double and triple labeled pixels; (iii) elevated frequencies of CD68+/CD163+/CD11c+ pixels are identified solely in renal cancers; (iv) across the three cancer types in most cases, densities of MC&M-associated pixels are greater in the tumor region compared to the surrounding stroma; (v) densities of CD68+ and CD163+ MC&M-associated pixel classes in tumor regions and in contact with CD3+ T cells are greater than densities of CD11b+ and CD11c+ pixel classes and (vi) distances of CD68+ and CD163+ pixel clusters to the edge of the tumor and to CD3 cells are shorter compared to CD11b+ and CD11c+ pixel clusters. Altogether, a pattern emerges demonstrating similarities in staining and spatial organization of MC&M-associated pixel populations across these three cancer types. However, the densities and spatial organization of CD68+ and CD163+ MC&M-associated pixel classes vs. CD11b+ and CD11c+ MC&M-associated pixel classes differ considerably.

Excellent recent reviews describe the complexity of MC&M subtypes in the tumor microenvironment (1, 2). The nomenclature of MC&M subtypes in human tumors is evolving as ontogenic and functional characteristics are being linked to phenotypes defined by expression of surface markers. A major distinction amongst tumor associated macrophages (TAMs) exists between M1, pro-inflammatory macrophages, identified by CD68+ staining, and M2 macrophages with pro-metastatic, immunosuppressive and pro-angiogenic activities that express CD163 and CD206 (43). In addition to TAMs, CD11b+ and HLA-DR-negative monocytic and granulocytic bone marrow derived myeloid cells infiltrate tumors and promote severe immune dysfunction (44). CD11c is expressed on dendritic cells. While stimulating tumor growth, metastasis and angiogenesis in an immature state, upon differentiation, CD11c+ macrophages acquire potent antigen presenting and anti-tumor functions (45). Dual antibody staining using anti- CD11b and anti-CD11c improved the subtyping of acute myelogenous leukemia and evaluation of treatment response with *in vivo* and *in vitro* correlations (46).

A mixed M1/M2 macrophage phenotype has been observed in kidney cancer (47), which in our profiling study also expresses abundant CD11c (Figure 4D). Our study demonstrates that this MC&M subtype is more abundant at the periphery of tumors and at a greater distance from T cells (Figures 6E, 7D). When comparing stromal and intratumoral MC&M-associated pixel densities, it appears that all pixel classes are increased inside compared to outside the tumor (Figure 6D), suggesting that tumors might exert mechanisms to attract cells marked by MC&M-associated pixel classes.

The PLEXODY staining assay that we piloted can be used with up to 10 antibodies per slide. Compared to manual or automated cyclic staining systems (48, 49), or to hyperplexed systems such as cyclic immunofluorescence (50), tissue mass cytometry (51), nCounter® applied to tissues and CODEX (52), the throughput of PLEXODY is greater. Up to 30 slides can be automatically stained with 5 antibodies using an overnight staining program. A major advantage of our method is the signal amplification through the tyramide amplification system used in the PLEXODY assay. While this extent of amplification may not be required for antibodies reactive with abundantly expressed CD antigens, when using the assay to measure the activation of signal transduction pathways or the localization of transcription factors, a highly amplified protocol is necessary (53).

At this time, the rate limiting step in the PLEXODY assay is the slide scanning. To optimize the efficiency of the workflow, we preselect regions for scanning. Scanning only regions that will be analyzed, significantly reduces the scanning time. However, this requires careful planning of data acquisition and analysis, since the IF signals are labile. While the mIHC component of the PLEXODY assay is permanent, the mIF is destroyed during the mIHC staining. Therefore, all the IF scanning and quality control has to be finalized before embarking on the IHC. When left untouched, the mIF slides are stable for weeks and months in the refrigerator, allowing to complete the mIF portion of the assay without a time constraint. After scanning, digital IF images provide a large dataset that can be reanalyzed to answer additional questions in future projects.

The application and proof-of-principle of the PLEXODY assay provided a deeper insight into the spatial organization of tumor associated MC&M-associated pixel classes. Previously published validation studies of the multiplex technology that included CD68 as a macrophage marker utilized a qualitative review of images to validate antibody staining patterns (28). We adopted the same approach that was used by these authors to demonstrate the staining specificity of our MC&M antibody panel (Figure 1D). Interesting, the outlines of CD68+ cells are identical in both studies, further confirming the antibody specificity and threshold settings of the anti-CD68 antibody reagent. The workflow of PLEXODY lies between the commercial multiplex staining and imaging with the OPAL/Vectra system that includes a proprietary staining kit and color deconvolution software and the open source system reported by Blom et al. (29). While the staining component of PLEXODY is automated, the data generation utilizes Matlab algorithms. We anticipate that the ability of the Matlab code for image co-registration and intersecting of single color pixel maps will be adopted in the future by one of the commercial analytics companies. This will allow our approach to be used for image analysis without requiring programming expertise.

In a different report, an extensive, quantitative and opened source immunophenotyping pipeline that is similar to our approach included a combination of 8 fluorescent and chromogenic antibodies, was applied to the same tissue section of a prostate cancer tissue microarray (29). Several important technical advances were accomplished in this study, such as whole slide co-registration of mIF and mIHC, nuclear segmentation and cell classification based on expression of CD45, CD4, CD8, FoxP3, Ki-67, androgen receptor, alpha-methyl-CoA racemase, cytokeratin 5, 8 and 18, E-cadherin and p63, and an integrative analysis of the entire multiplexed dataset from the whole slide. While this study is based on nuclear segmentation of lymphocytes, the approach in our study is based on a pixel based segmentation of myeloid cells and macrophages. To generate the T cell mask, we also apply the nuclear segmentation method. Choosing the right method to generate cellular masks depends on the morphology of cells. Cells with a dendritic morphology (macrophages, neurons, mesenchymal cells) require selection of an appropriate image analysis method to capture the signal from cellular processes.

A systems pathology approach was applied by Bruck et al. (54) to demonstrate the relationship between treatment response and cellular organization in the tumor microenvironment of chronic myelogenous leukemia. Integrative data analysis using quantitative measurements of slides stained with a fluorescent 5-plex and chromogenic 3-plex revealed an immunosuppressive tumor microenvironment and adverse outcomes for patients with a low percentage of CD4+ and high percentage of PD1+/TIM3-/CD8+ T cells (54). Including data from mIF/mIHC measurements in the outcomes prediction model outperformed conventional stratification by BCR-ABL PCR-based quantification (54).

Another example of the power of the multiplex analytical approach is demonstrated in a study of lung cancer biopsies (32). Distances between tumor and CD8+ cells and ratios of cytotoxic-to-regulatory T cells predicted response to treatment and patient outcomes. Another recent, elegant study in Hodgkin's lymphoma provided a detailed mapping of distances between tumor associated macrophages, labeled with CD68 and CD4+/CD8+ T cells (55). Measurements of PD-1 and PD-L1 expression were also included in this study providing important, treatment related information. Further, the authors demonstrated specific cell-cell interactions by measuring Euclidian distances between cell populations. The data from this study revealed that PD-L1+/CD68+ TAMs reside in the vicinity of T cells and that CD4+ T cells expressing PD-1 aggregate around Reed Sternberg cells (55).

There are several limitations of our study. Unfortunately, we were not able to determine the actual cell numbers of MC&Ms. The numbers of pixels associated with each cell may vary and counting macrophages, which are 20–80 microns in length, requires a 3-dimensional visualization technique. This can potentially be accomplished with tissue clearing protocols (56) and imaging via confocal or light sheet microscopes (57). However, these approaches are low throughput and difficult to standardize. Another limitation is the thresholding of the fluorescent images. While we tested automated thresholding systems, we could not find one that reproduces the manual thresholding. Therefore, thresholds need to be optimized by the laboratory performing the image analysis. Finally, another limitation is the dependence of the signal intensity on the amplification reagents. The commercial secondary antibody we used is conjugated to a high numbers of horse radish peroxidase (HRP) molecules. Other vendors may differ in the ratio of antibody to HRP molecules, which directly effects the brightness of the stain. Thus, while the method and PLEXODY pipeline remains the same when using reagents from other sources, percentages of cell populations may decrease due to loss of low intensity pixels.

In summary, we demonstrate the feasibility of an automated staining process, combined with image co-registration, digital image analysis and data integration from whole slides. Modules of multiplex staining, image acquisition and processing and data extraction and analysis are combined to provide a high content dataset of MC&M-associated pixel classes in the tumor microenvironment. As demonstrated in other studies, measurements of immune cell densities and distances provide important insights into cellular interactions before treatment, are predictive of treatment responses. In addition, the spatial organization of immune cells was shown to be affected by cytokines that are released by macrophages or cancer cells (58–60). While we applied the PLEXODY assay to profile MC&M-associated pixel classes, it can also be used for measurements of pathway activation and transcriptional activity by using different panels of antibodies. Thus, due to its efficiency and automation, the PLEXODY assay has broad applicability for translational research studies that consist of large cohorts of patients.

## Author Contributions

JS, ZM, and BK: Generated data. JS, ZM, HG, FH, and AG: Analyzed data. HG, LW, SS, and AG: Data interpretation. HG, AV, SP, NN, AG, AC, and BK: Manuscript writing.

### Conflict of Interest Statement

The authors declare that the research was conducted in the absence of any commercial or financial relationships that could be construed as a potential conflict of interest.
