# Supplementary material for: Spatial Mapping of Myeloid Cells and Macrophages by Multiplexed Tissue Staining
Source: Front Immunol. 2018 Dec 14;9:2925. doi: 10.3389/fimmu.2018.02925 (PMC6302234; doi:10.3389/fimmu.2018.02925)

Supplementary Figure 1

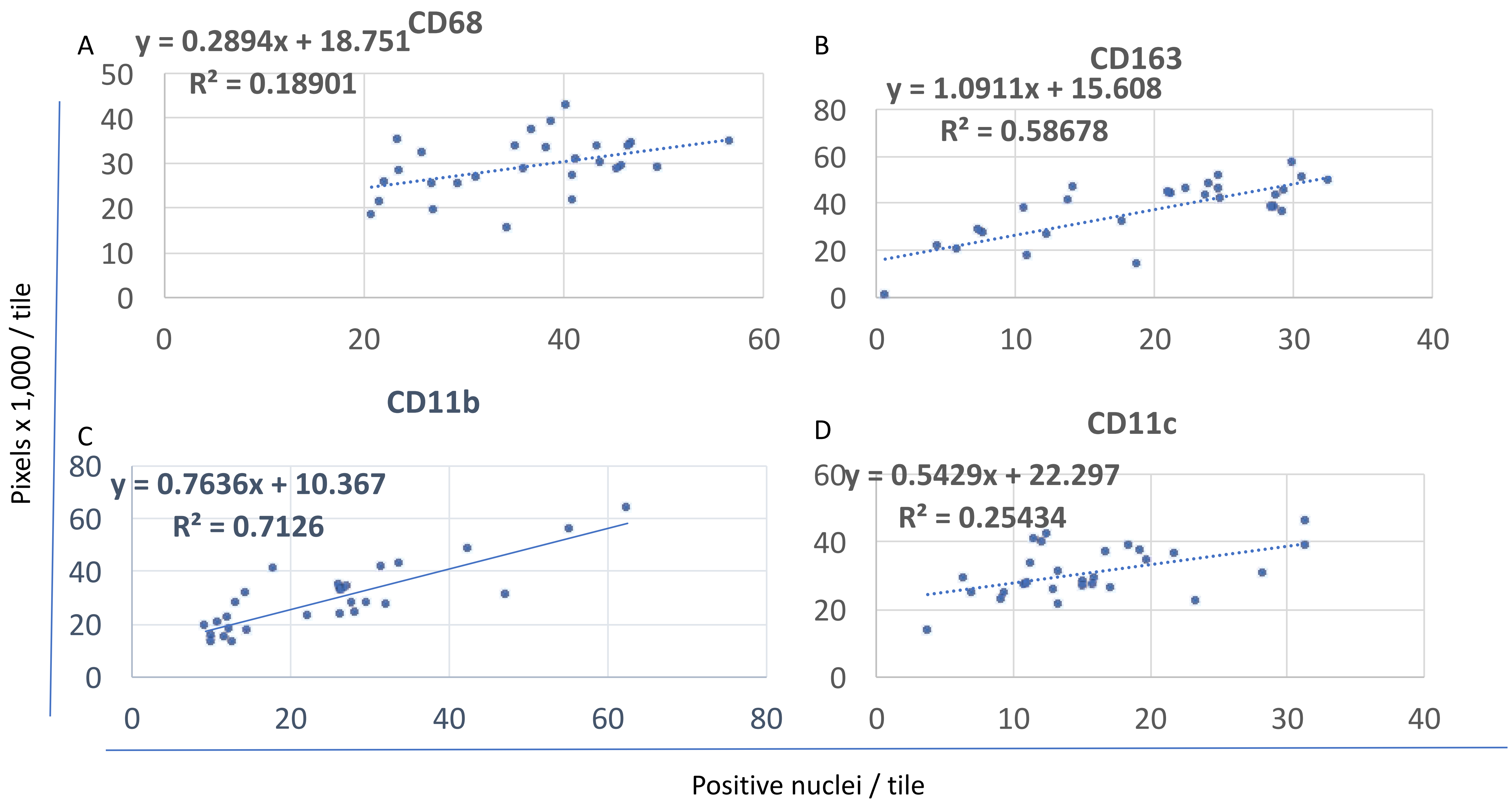

Supplementary Figure 1E

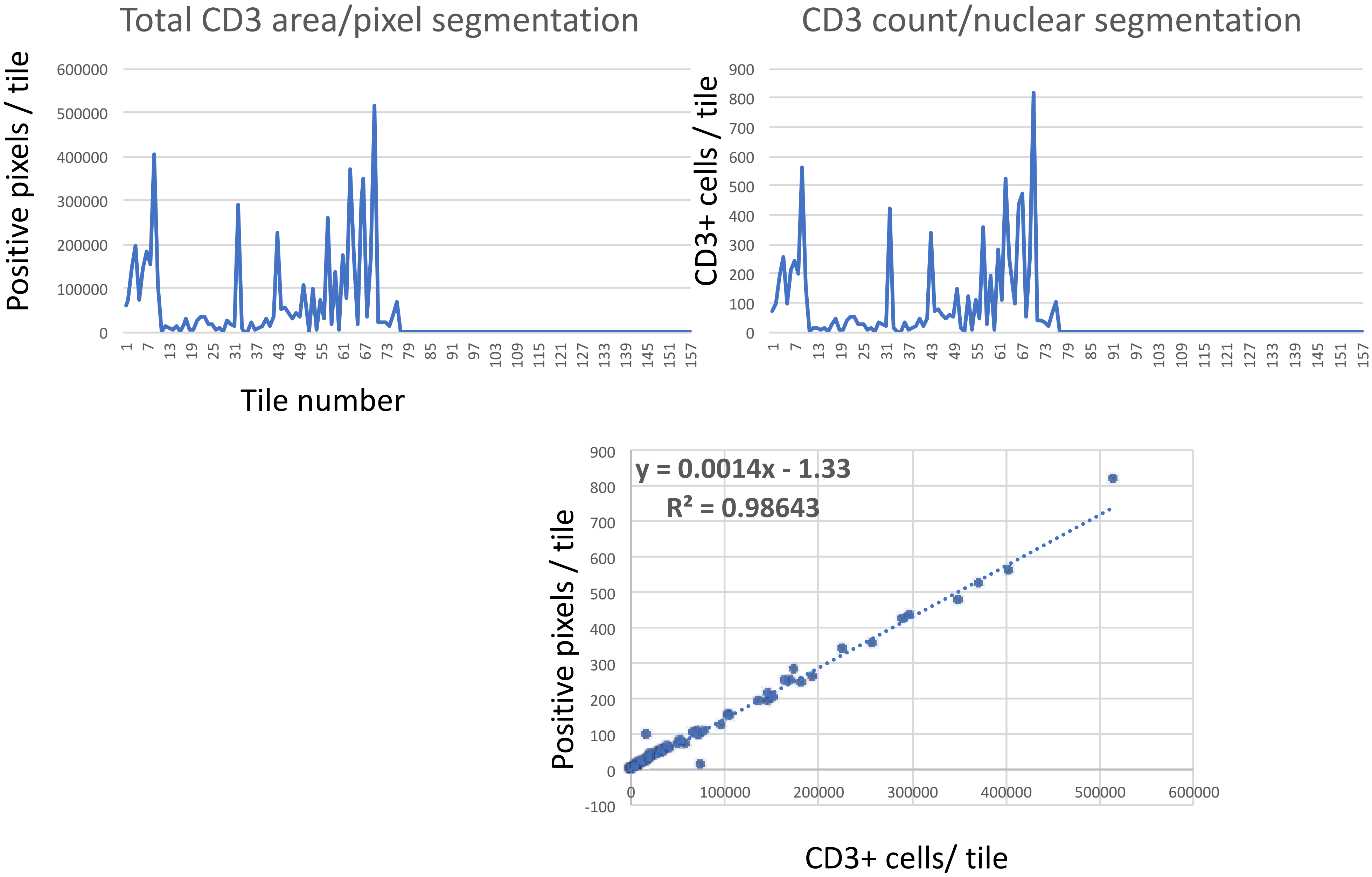

Supplementary Figure 2, tonsil

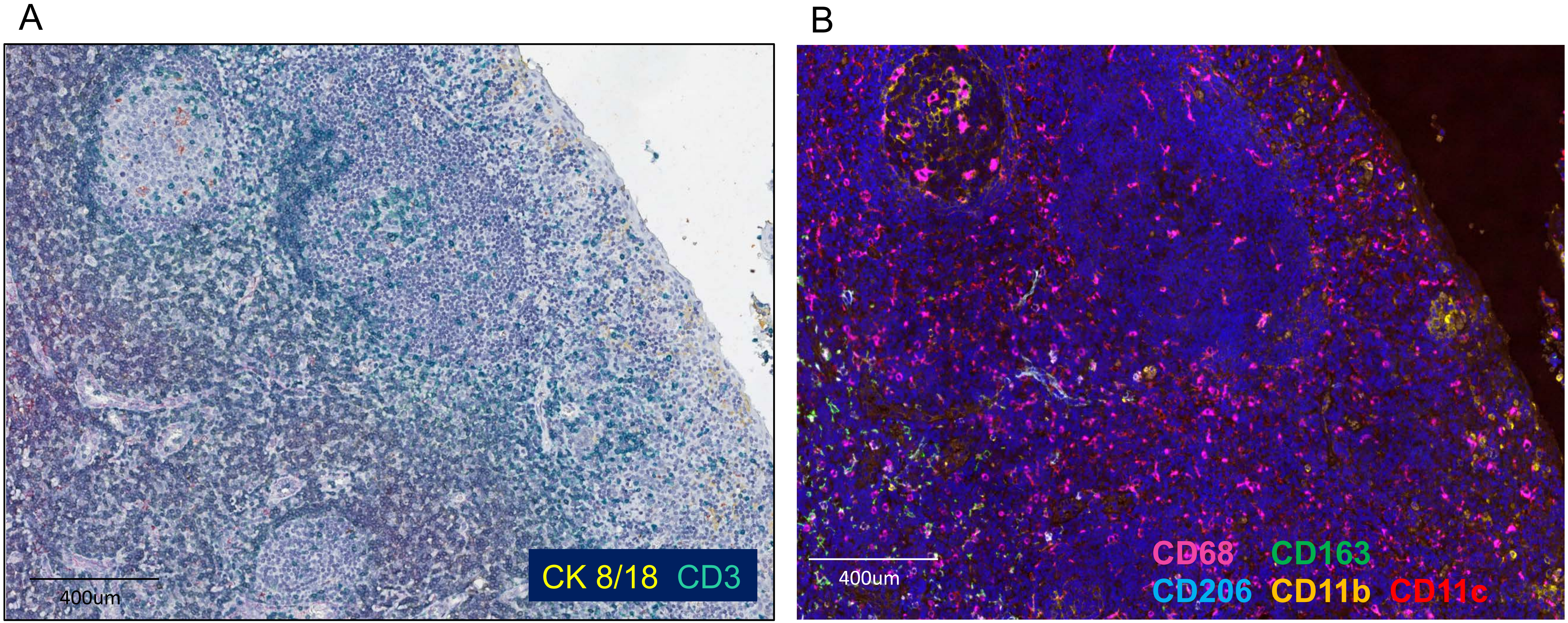

Supplementary Figure 3, inflamed colon

A

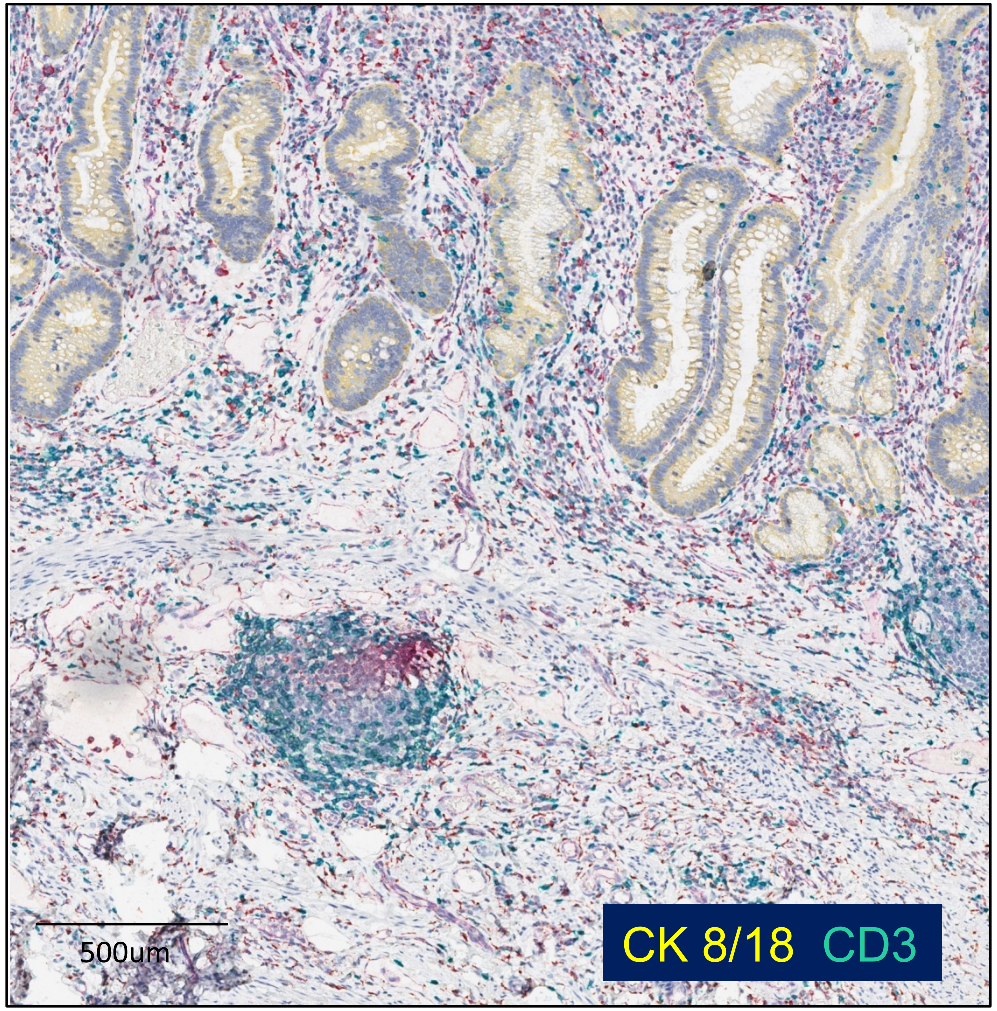

B

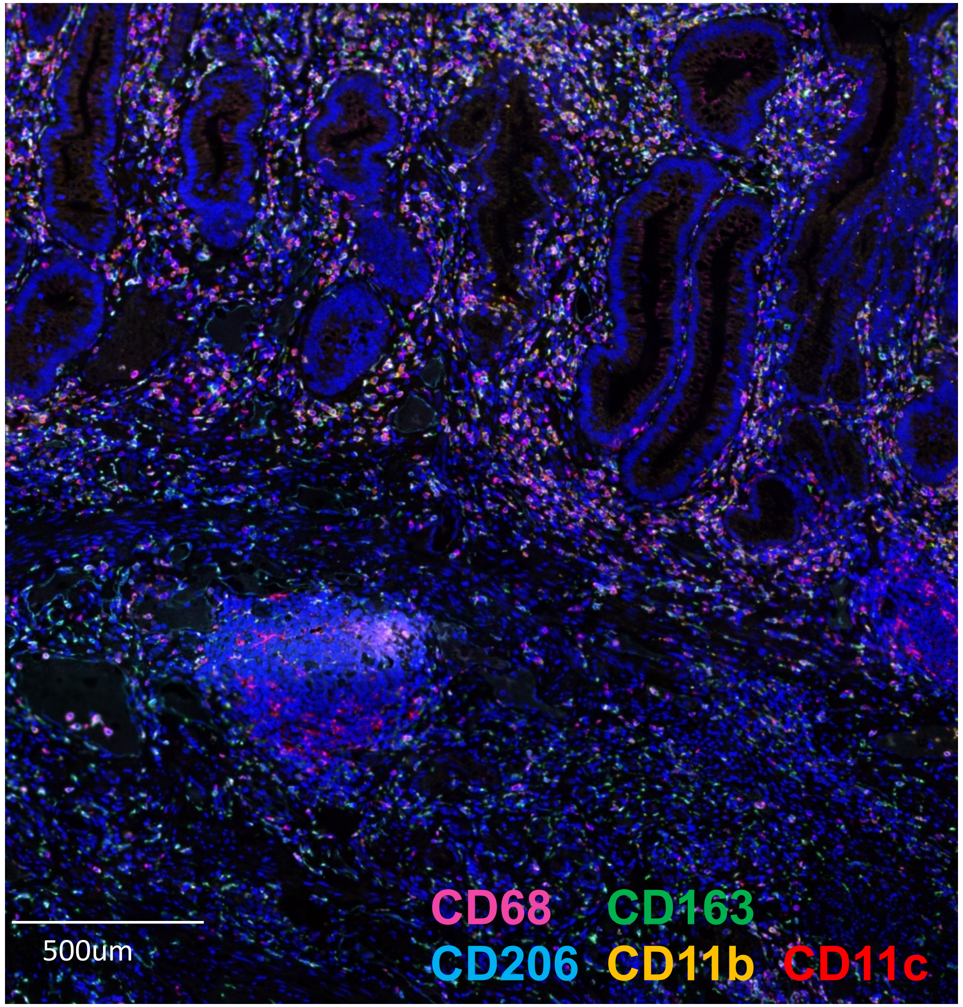

Supplementary Figure 4, pancreatic cancer

A

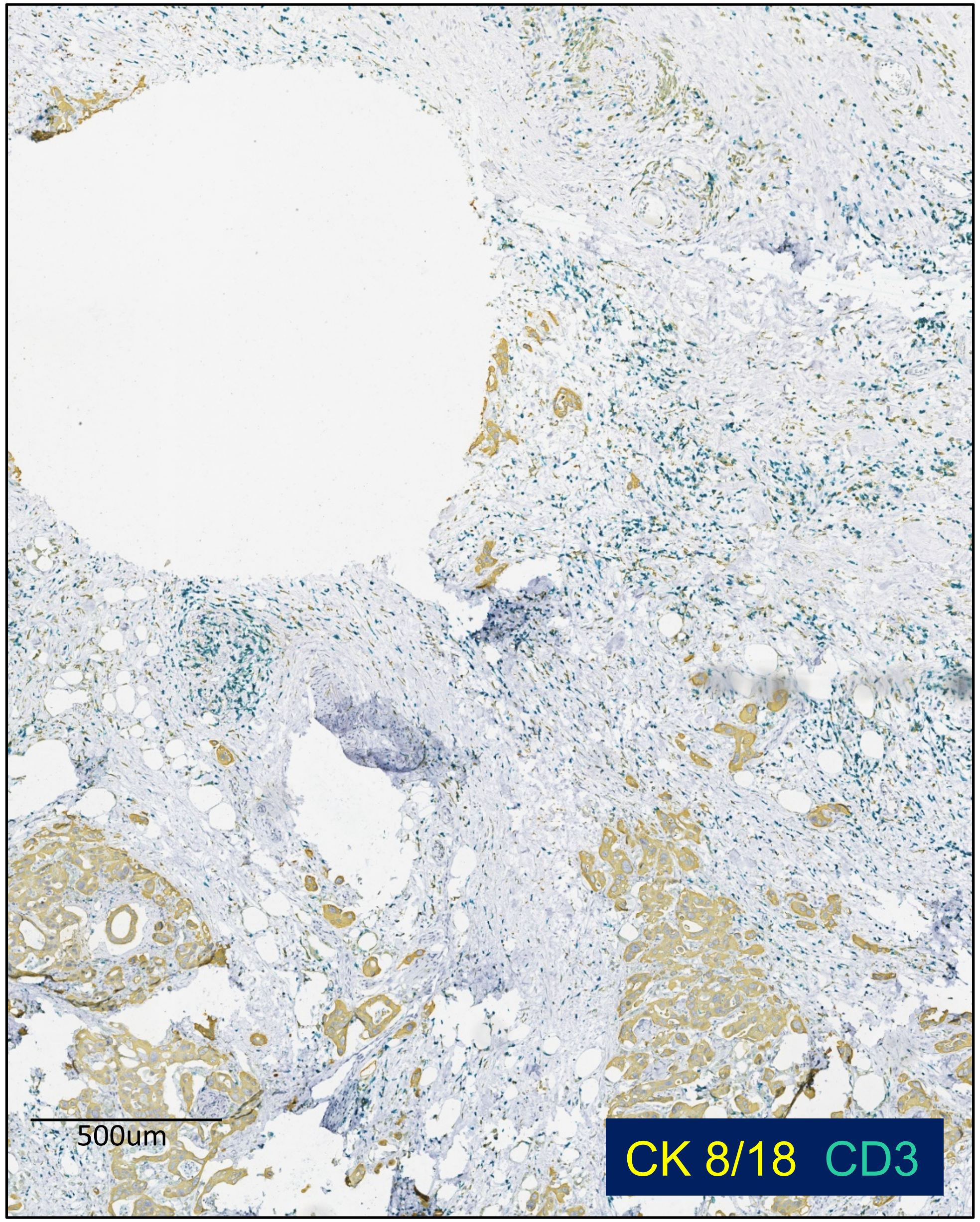

B

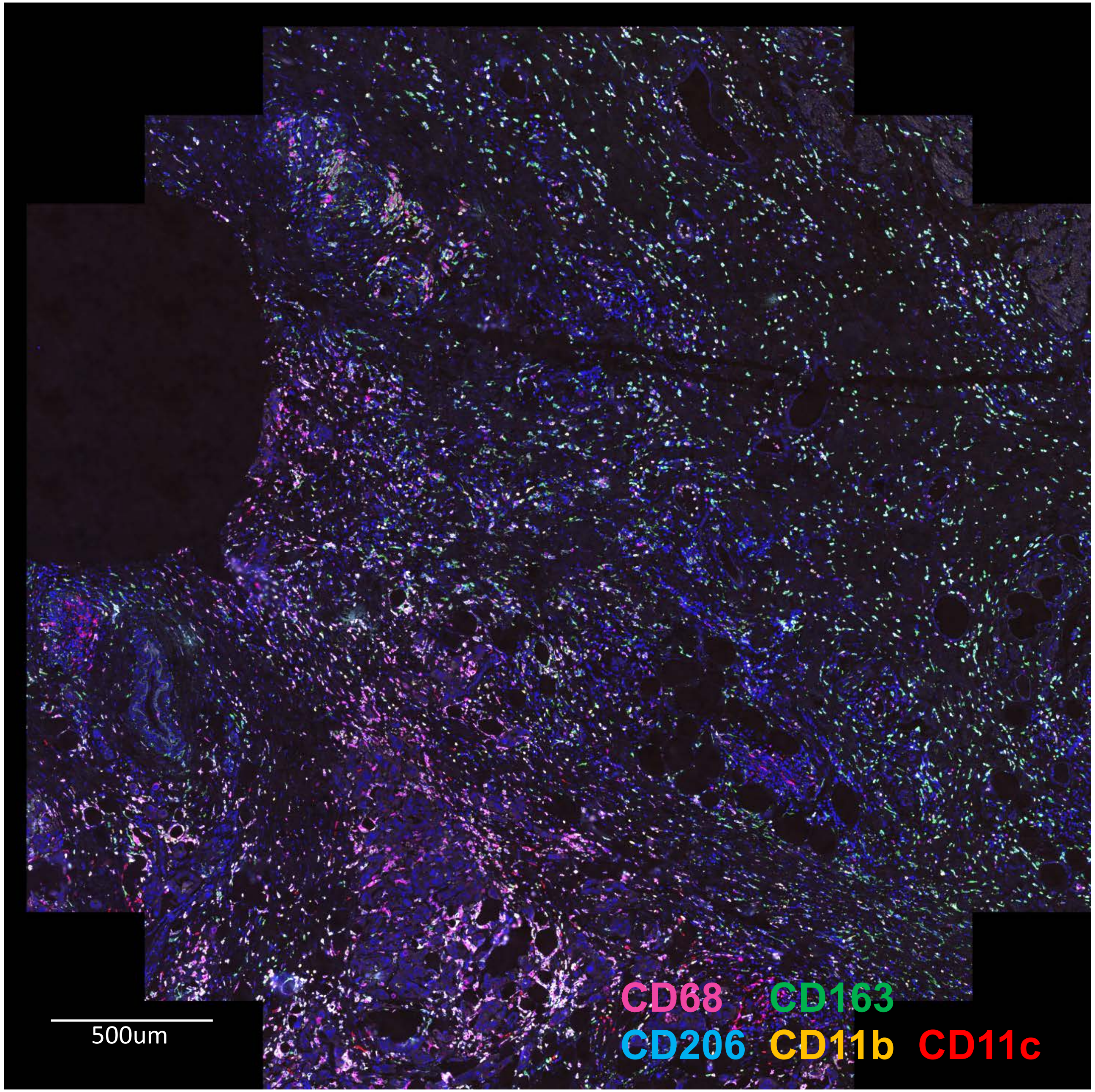

Supplementary Figure 5, pancreatic cancer

A

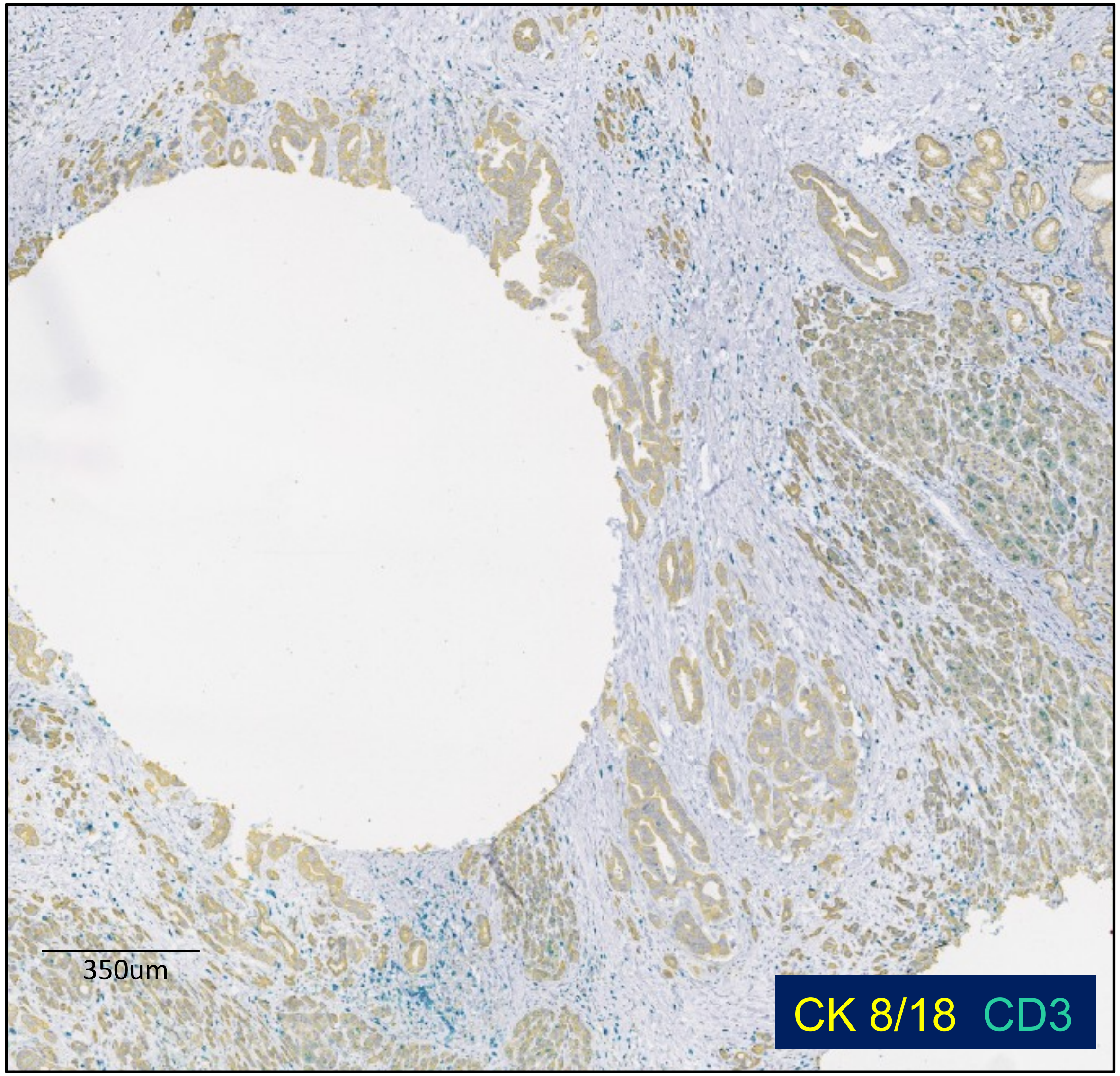

B

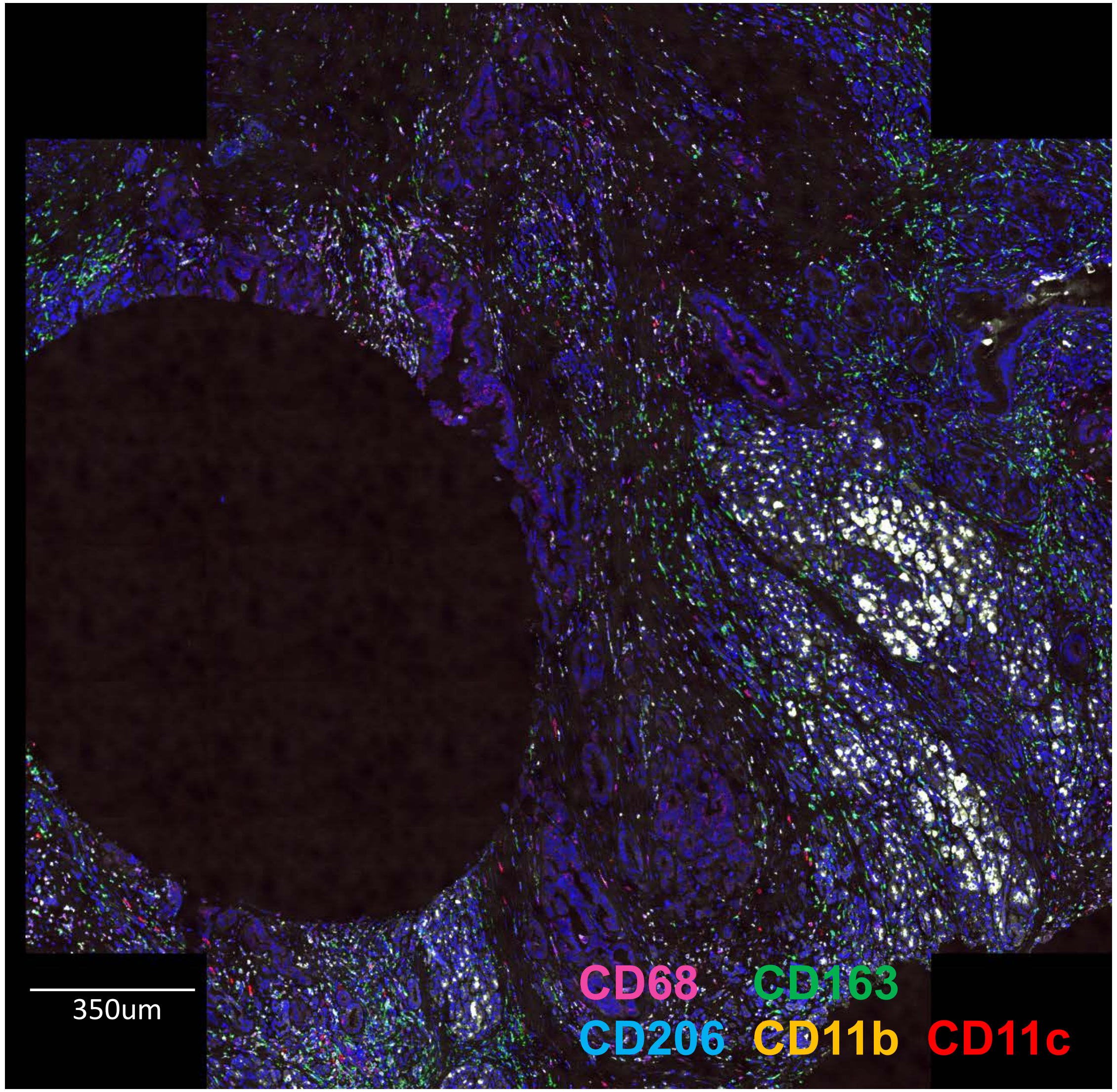

Supplementary Figure 6, pancreatic cancer

A B

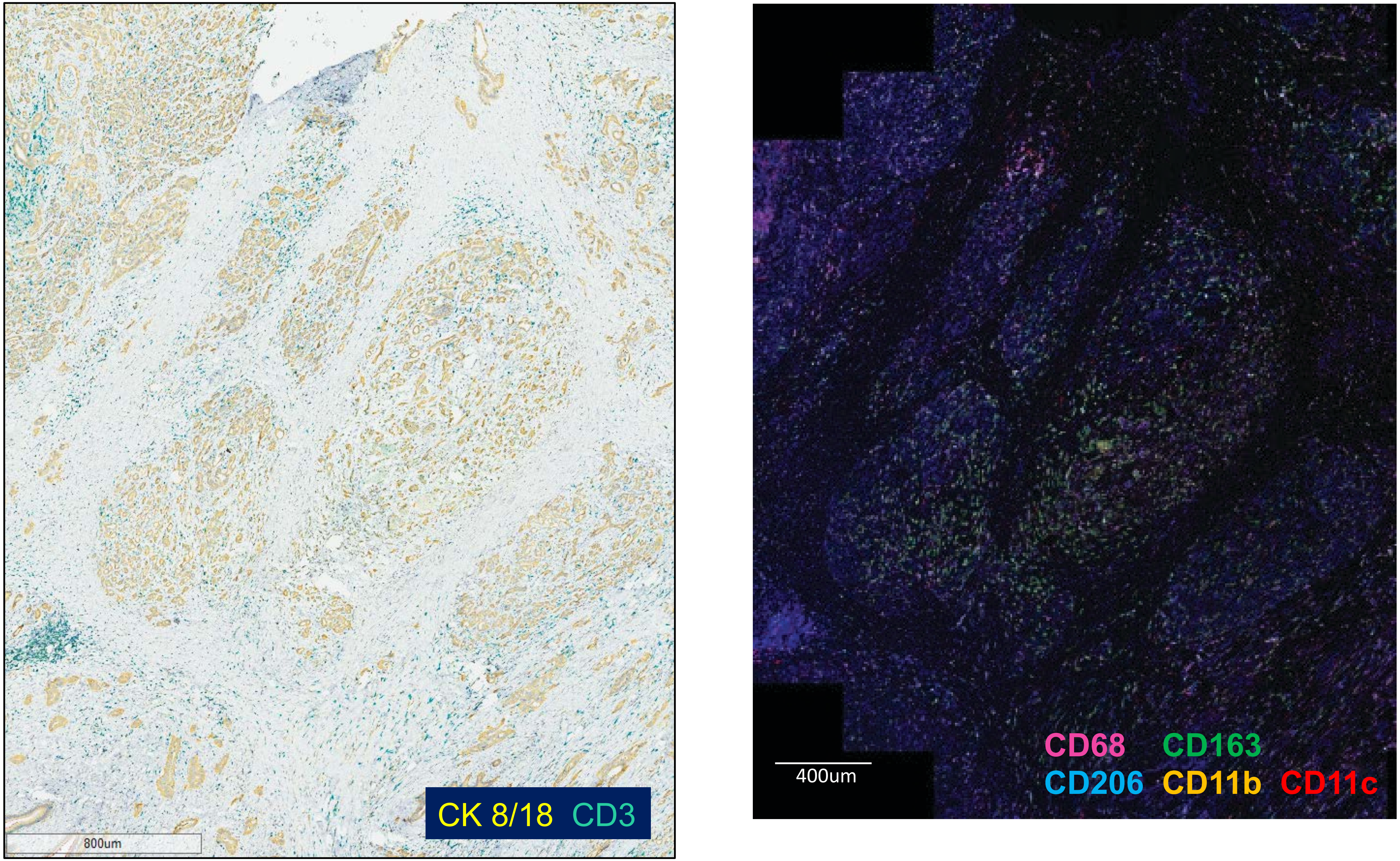

Supplementary Figure 7

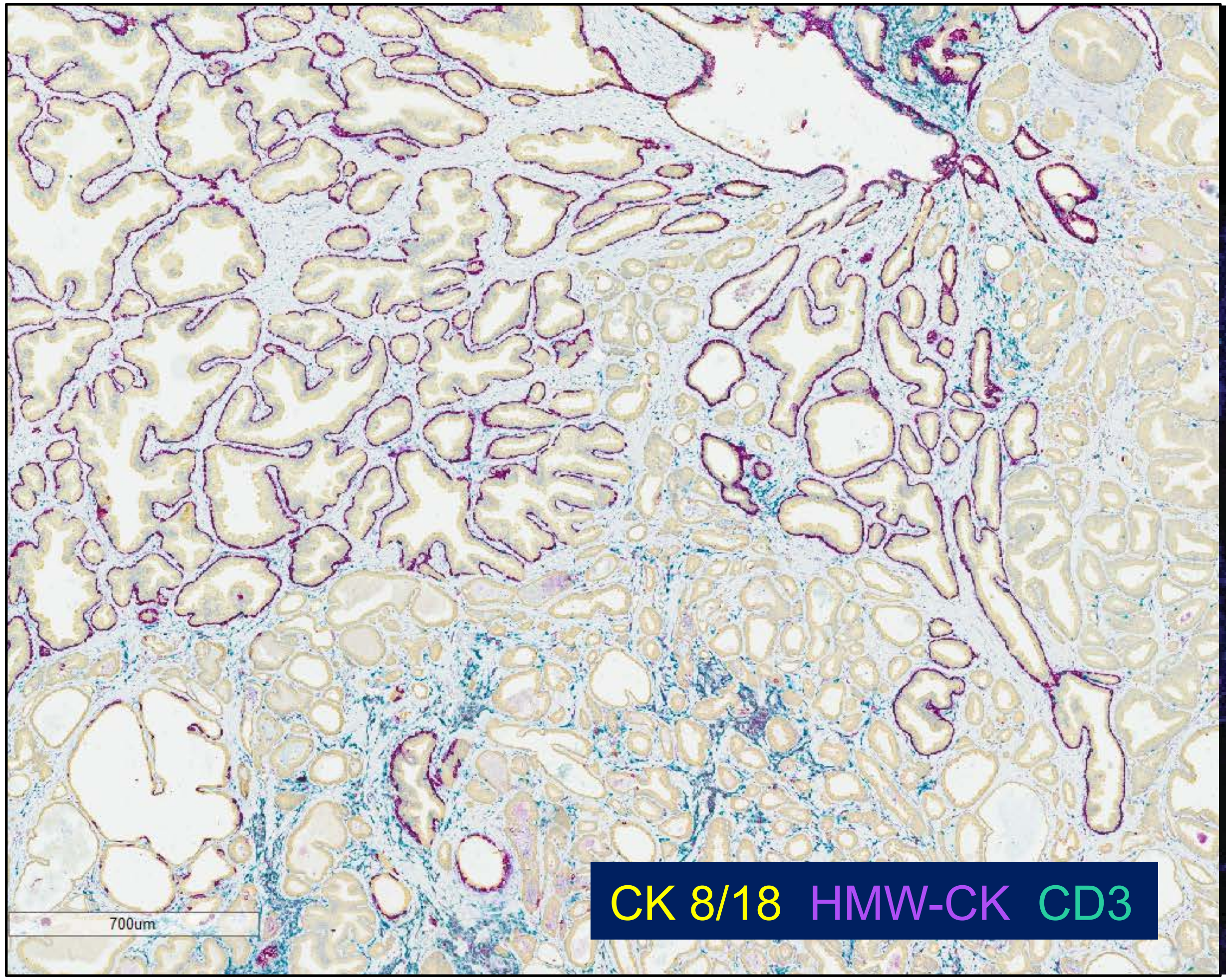

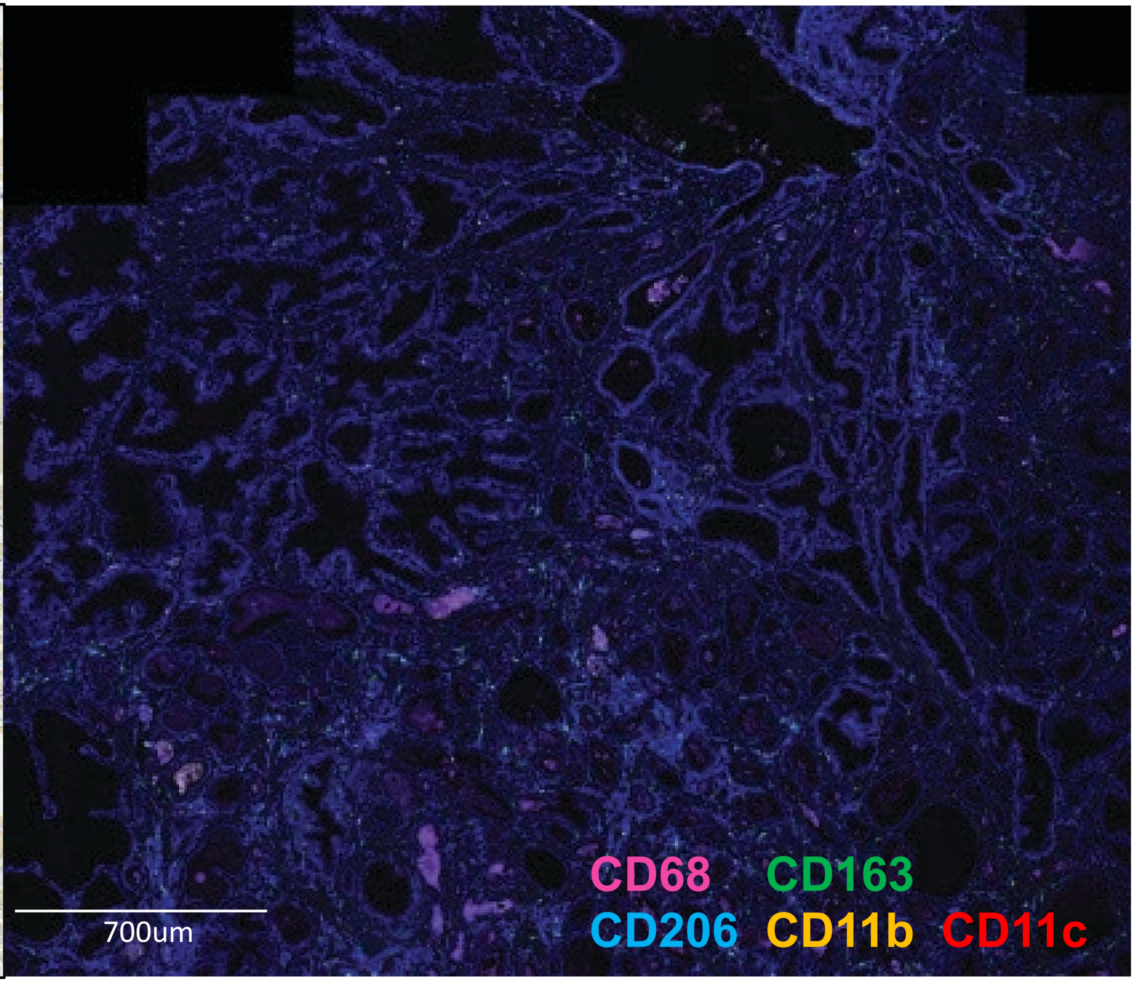

Supplementary Figure 8, prostate cancer

A

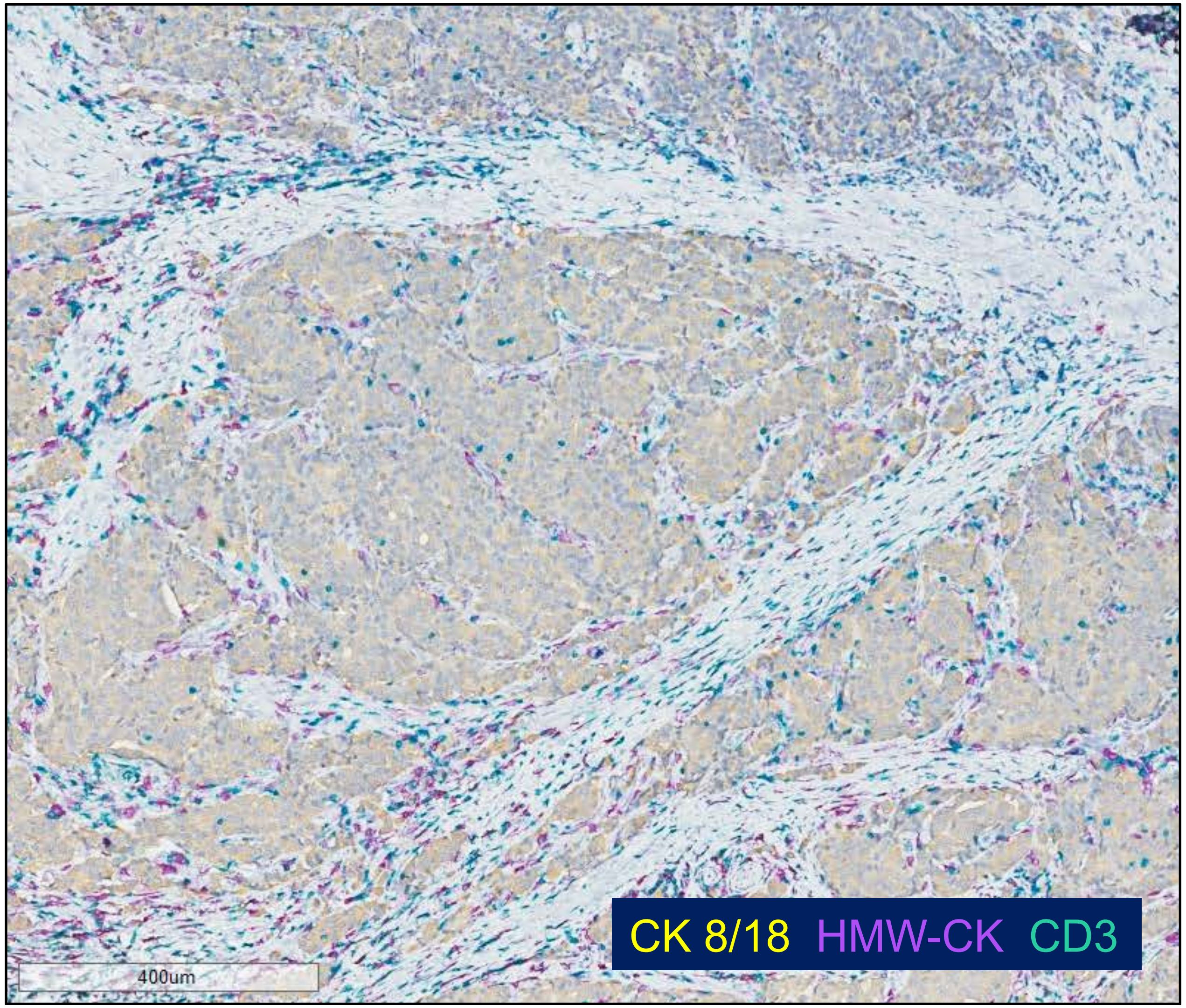

B

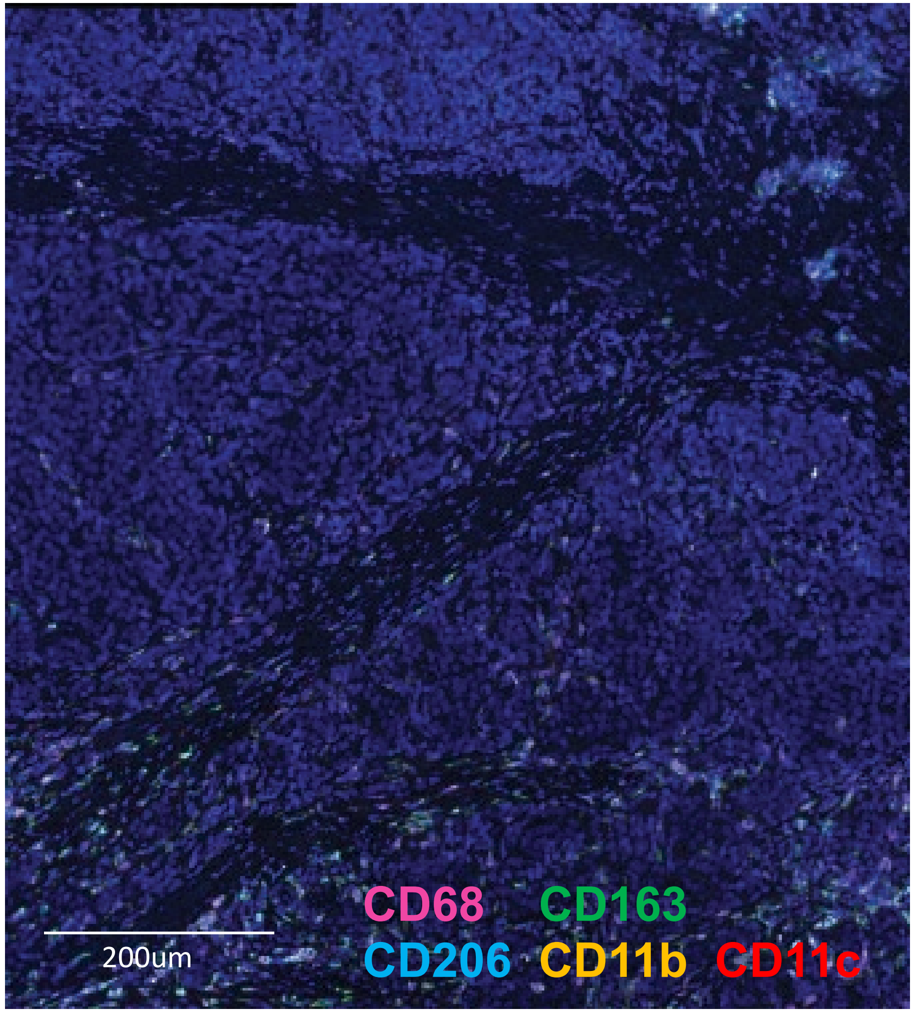

Supplementary Figure 9, prostate cancer

A

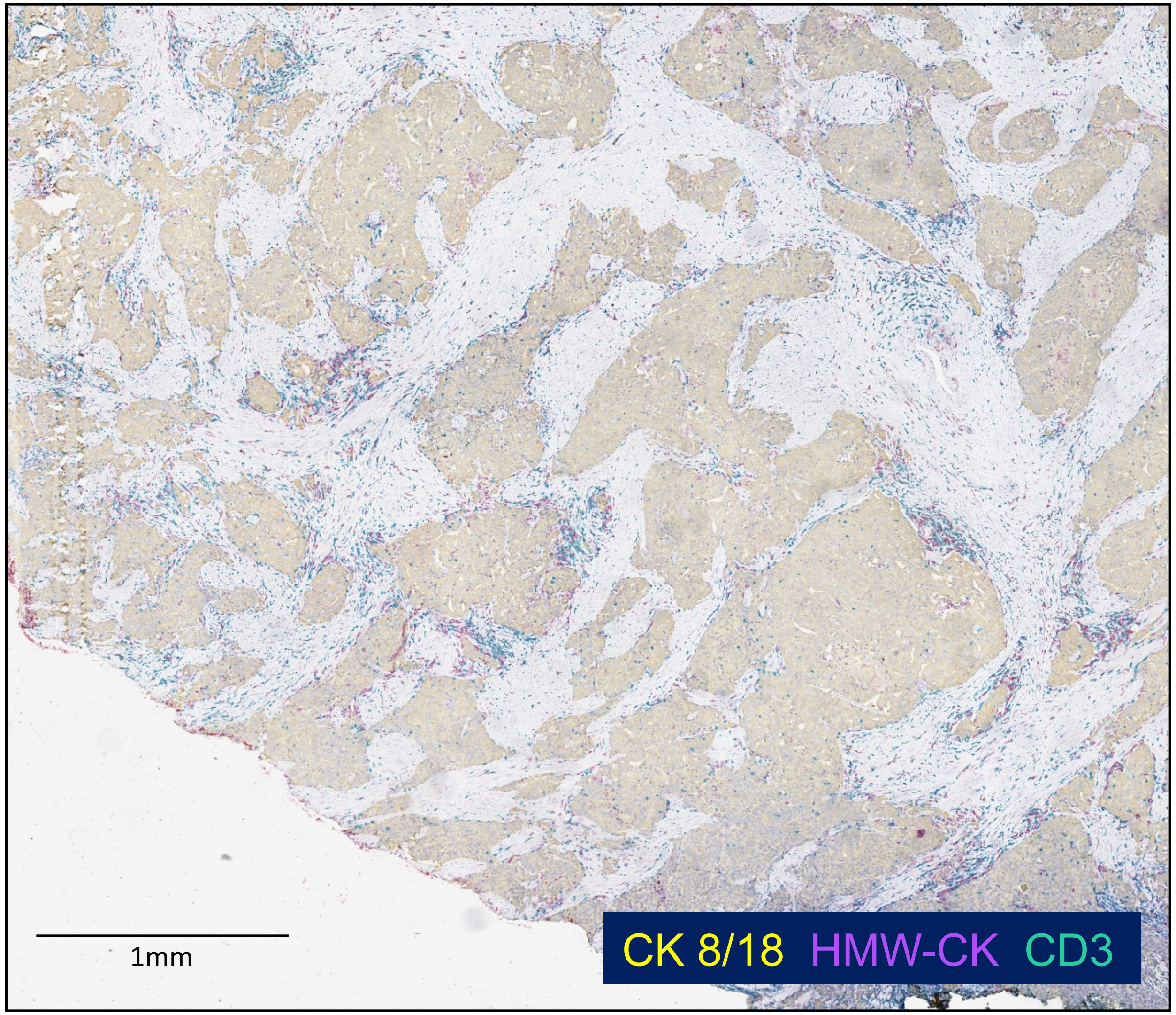

B

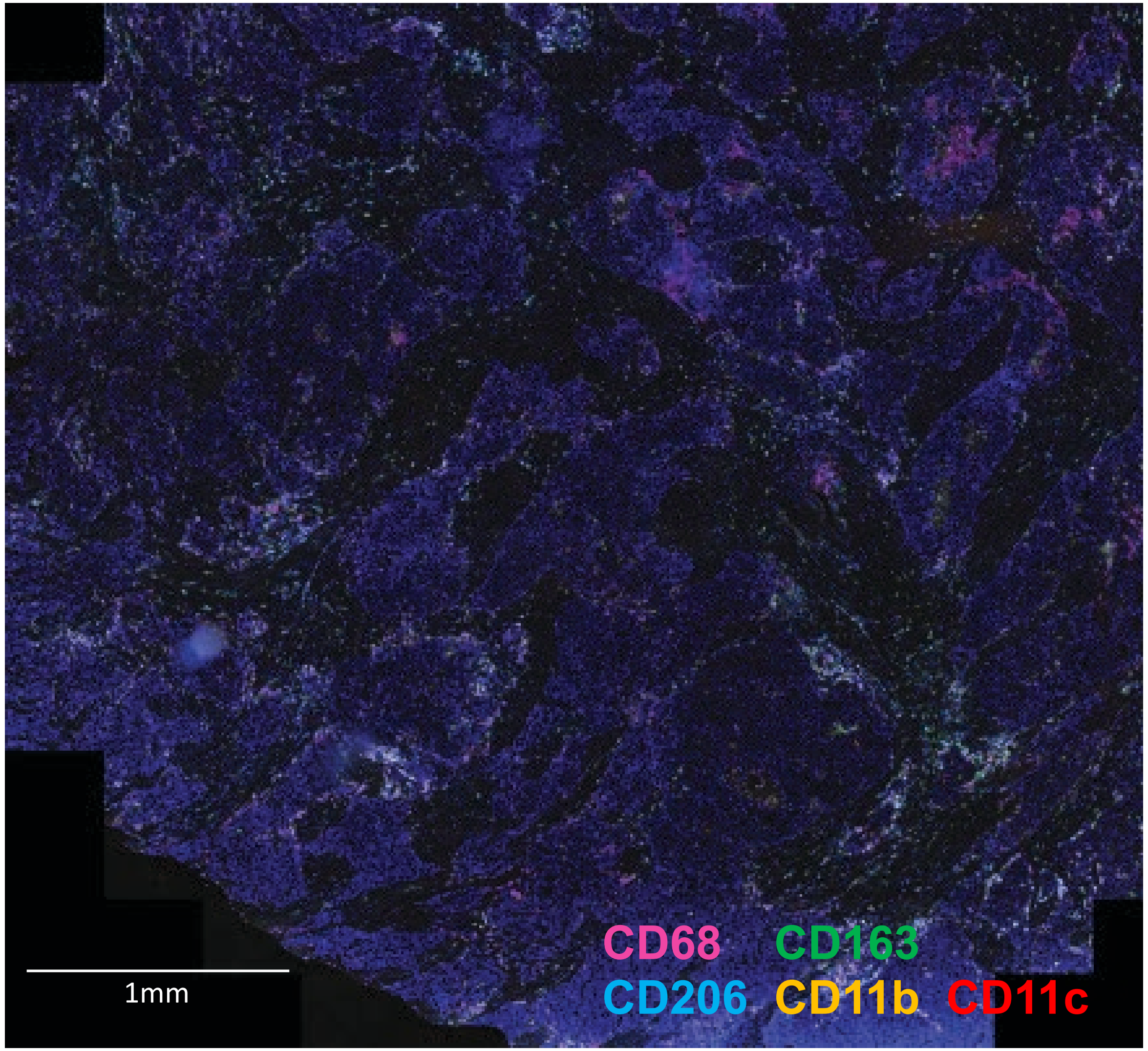

Supplementary Figure 10, renal cancer

A

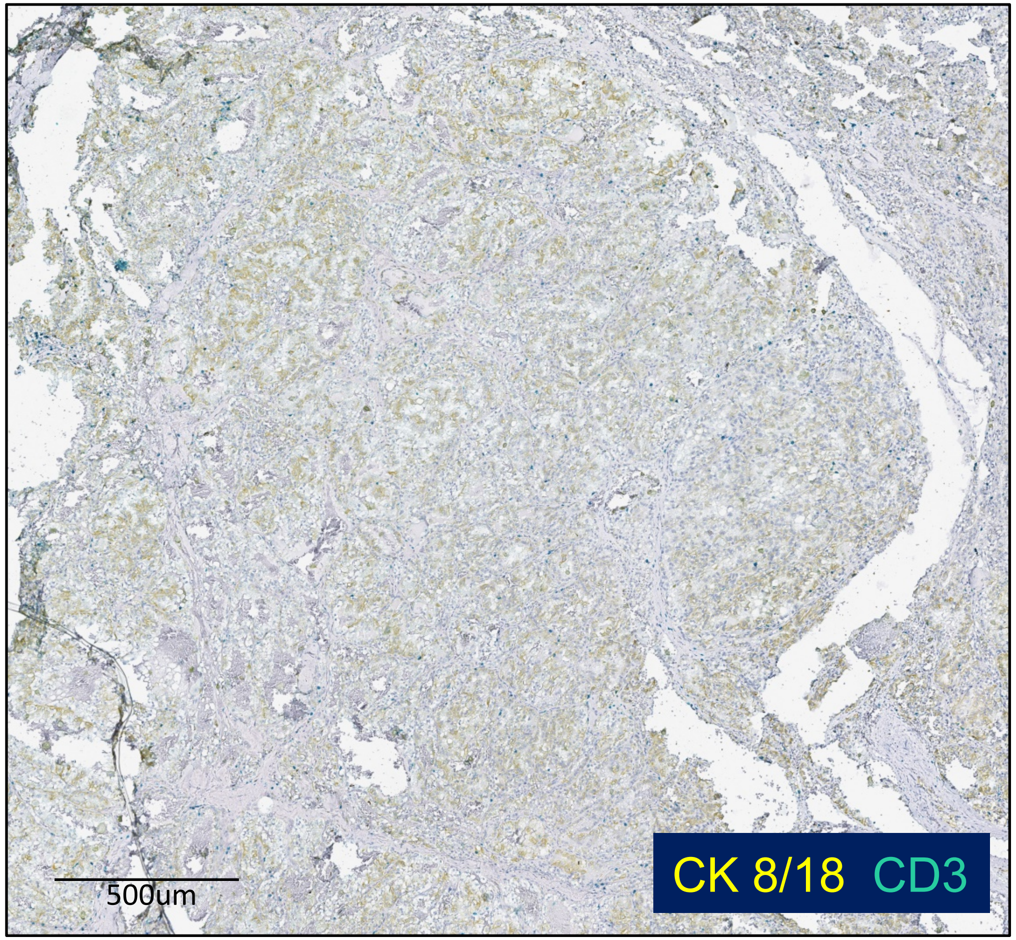

B

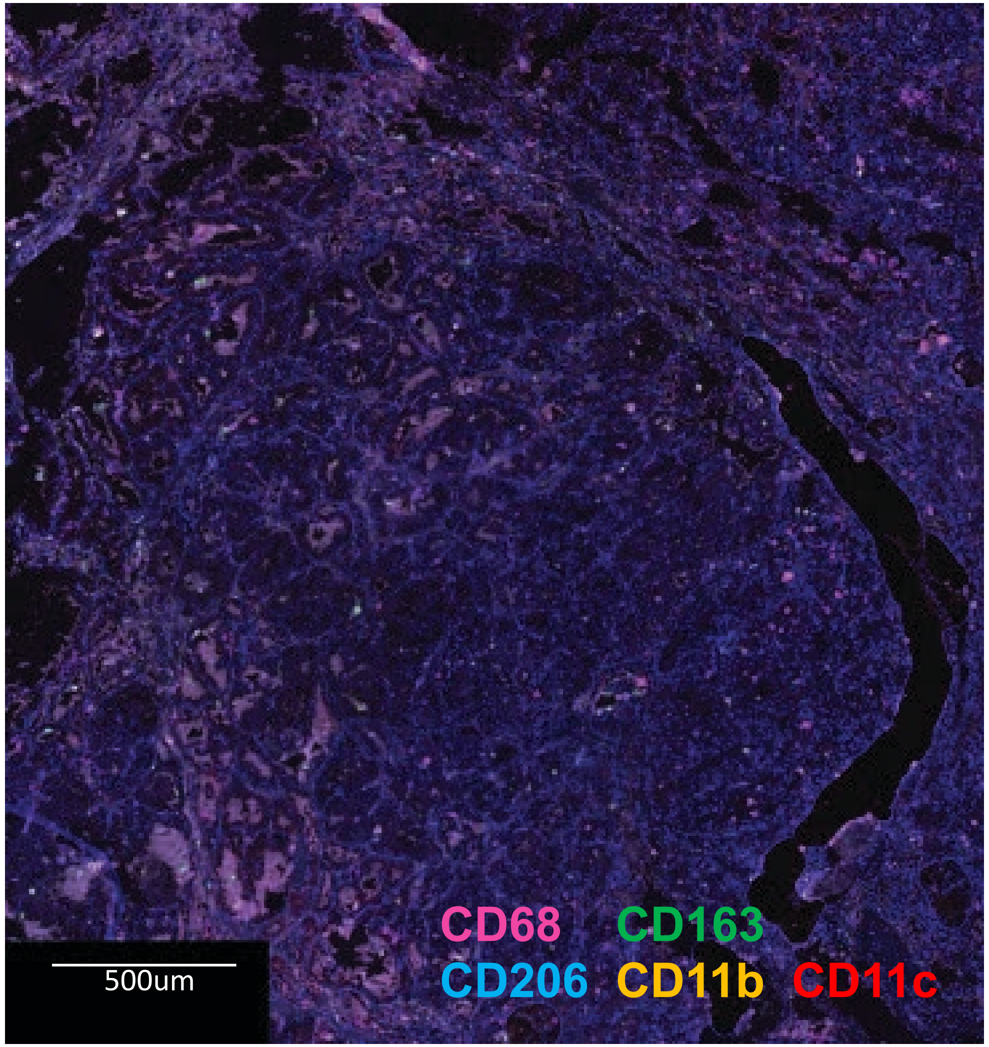

Supplementary Figure 11, renal cancer

A

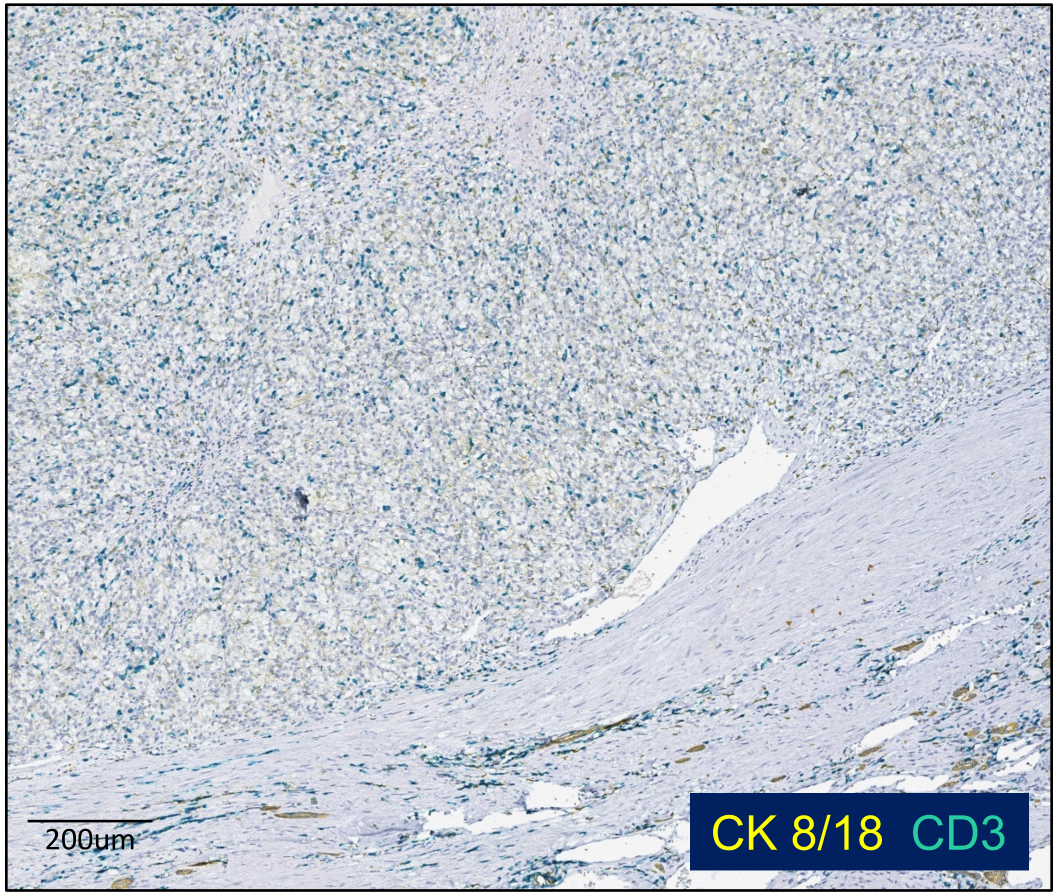

B

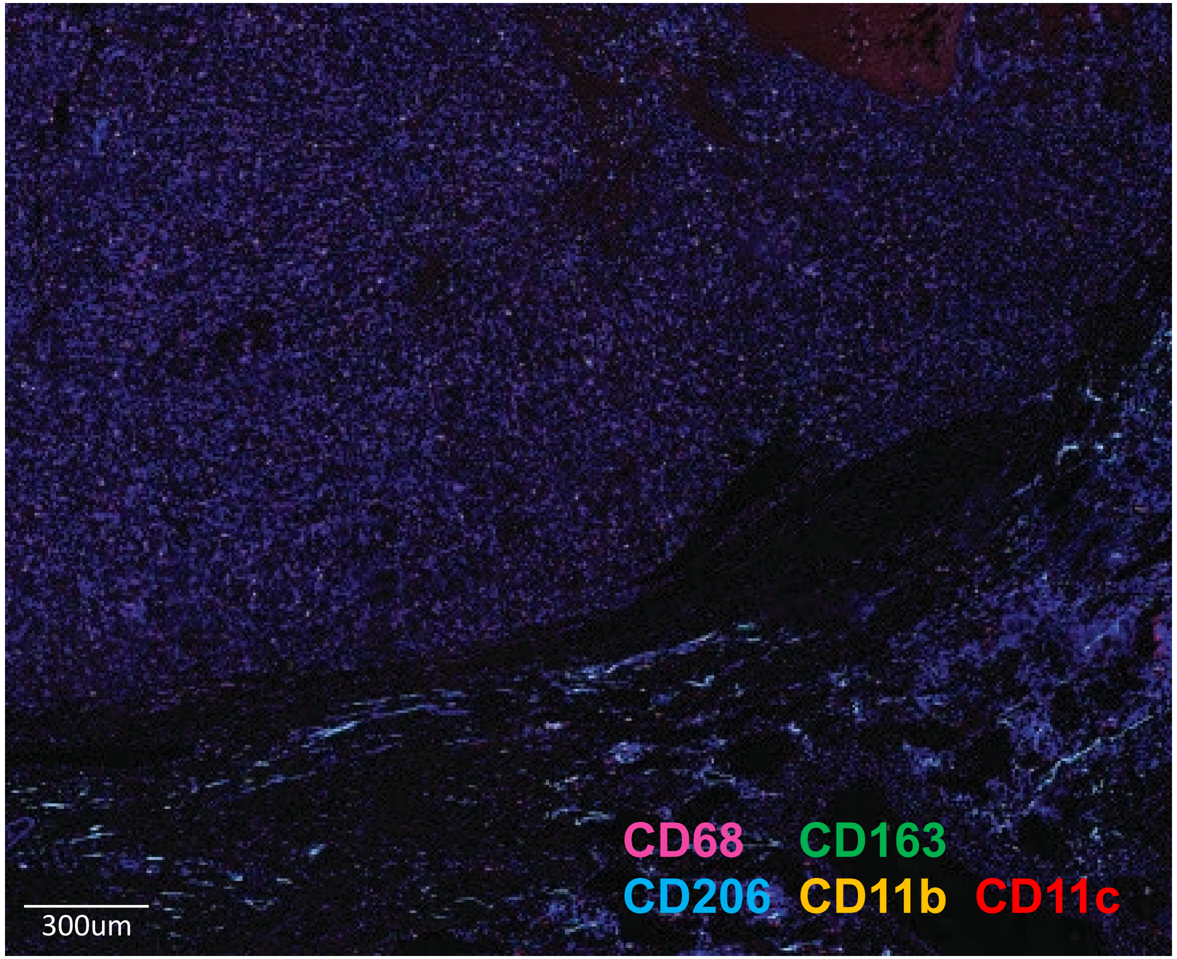

Supplementary Figure 12, renal cancer

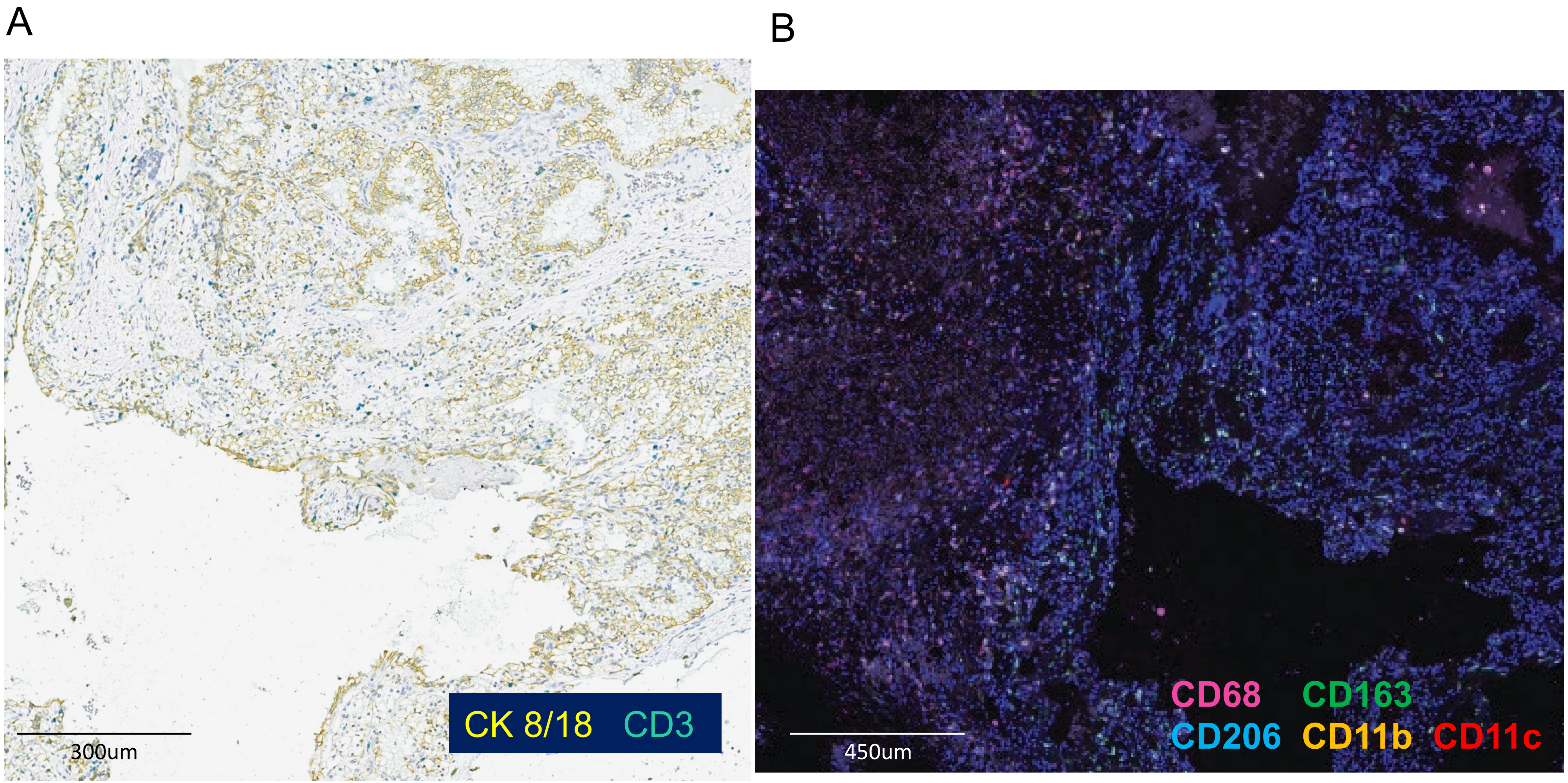

Supplement: Supplementary file 1 [file Data_Sheet_1.pdf]
