# Supplementary material for: Spatial Mapping of Myeloid Cells and Macrophages by Multiplexed Tissue Staining
Source: Front Immunol. 2018 Dec 14;9:2925. doi: 10.3389/fimmu.2018.02925 (PMC6302234; doi:10.3389/fimmu.2018.02925)
Supplement: Supplementary file 2 [file Data_Sheet_2.PDF]

Supplementary Tables

**Supplementary Table 1.** Antibody staining intensities after 1 and 5 antigen retrieval treatments										
	CD11b 1 Ret	CD11b 5 Ret	CD11c 1 Ret	CD11c 5 Ret	CD68 1 Ret	CD68 5 Ret	CD163 1 Ret	CD163 5 Ret	CD206 1 Ret	CD206 5 Ret
values	0.259	0.233	0.216	0.241	0.229	0.254	0.239	0.269	0.252	0.269
StDev	0.127	0.131	0.127	0.144	0.179	0.220	0.157	0.166	0.157	0.166
p-value (1Ret/5Ret)		0.556		0.983		0.713		0.323		0.664

**Supplementary Table 2.** Colon mucosa: % of MC&M-mask					
antibody	CD68	CD163	CD11b	CD11c	P1+
%	11.660	15.123	20.708	11.832	35.732
StDev	5.241	6.425	11.159	5.863	8.072

**Supplementary Table 3.** Tonsil germinal center: % of MC&M-mask					
antibody	CD68	CD163	CD11b	CD11c	P1+
%	15.33	1.80	45.39	20.95	16.31
StDev	6.23	1.96	14.05	7.14	4.65

**Supplementary Table 4.** % single positive MC&M subtypes (**Figure 4A**)										
		Panc 1 n=6	Panc 2 n=8	Panc 3 n=9	Prost 1 n=9	Prost 2 n=7	Prost 3 n=9	Kid 1 n=8	Kid 2 n=10	Kid 3 n=10
CD68*	Average	62.710	10.843	19.703	61.939	14.611	15.346	34.661	22.921	6.539
	StDev	16.519	6.019	11.303	15.679	10.134	9.229	12.668	10.229	5.818
CD163	Average	25.386	34.798	40.310	7.798	20.167	46.555	9.873	27.334	54.070
	StDev	14.664	18.726	17.120	5.072	16.115	20.745	3.536	8.794	11.867
CD11b	Average	1.396	11.002	5.307	2.771	7.834	2.119	1.935	5.268	7.522
	StDev	1.118	6.742	3.529	4.499	3.670	1.785	1.019	4.041	4.560
CD11c	Average	0.779	3.829	6.344	0.703	0.648	3.497	26.712	4.047	3.198
	StDev	1.186	3.051	7.202	0.921	0.451	4.342	16.179	1.818	2.634
**P1+	Average	9.729	39.528	28.336	26.790	56.740	32.483	26.820	40.430	28.671
		5.579	16.310	5.736	7.111	19.000	14.642	6.447	7.203	6.192

*Numbers are the percent of the M-mask.
** The P1 population is calculated by subtracting all single color pixels from the total MC&M-mask (unity of pixels).

**Supplementary Table 5.** % double positive MC&M subtypes (**Figure 4B**)										
		Panc 1 n=6	Panc 2 n=8	Panc 3 n=9	Prost 1 n=9	Prost 2 n=7	Prost 3 n=9	Kid 1 n=8	Kid 2 n=10	Kid 3 n=10
CD68_ CD163	Avg	91.932	32.858	77.761	74.709	40.477	55.722	57.647	73.445	56.563
	Stdev	6.848	10.261	15.909	23.122	24.331	18.465	14.035	8.957	11.542
CD68_ CD11b	Avg	1.492	2.111	1.089	15.790	1.351	0.296	0.760	2.838	2.234
	Stdev	0.908	1.784	0.812	22.246	0.763	0.332	1.405	2.022	2.804
CD68_ CD11c	Avg	0.930	1.222	5.524	1.157	0.490	0.714	19.266	7.686	2.162
	Stdev	1.287	1.767	11.658	0.781	0.367	0.943	12.163	4.556	2.687
CD163_ CD11b	Avg	0.174	2.292	0.864	0.125	1.836	0.647	0.005	0.226	0.870
	Stdev	0.124	2.161	0.934	0.164	1.830	0.905	0.013	0.140	1.107
CD163_ CD11c	Avg	0.935	2.049	1.313	0.049	0.224	0.675	3.493	3.501	1.343
	Stdev	1.089	3.780	1.331	0.048	0.281	0.882	1.771	1.641	1.261
CD11b_ CD11c	Avg	0.041	1.411	0.642	0.265	2.053	0.369	0.707	0.362	0.900
	Stdev	0.059	1.272	0.903	0.432	2.480	0.617	0.692	0.402	0.734
**P2+	Avg	4.496	58.057	12.806	7.904	53.569	41.578	18.122	11.943	35.927
	Stdev	4.460	13.555	10.377	6.024	21.825	17.448	7.937	3.809	9.913

*Numbers are the percent of P1+ pixels listed in Supplementary Table 4. Double positive pixels are obtained directly from images as the unity of the two antibody masks. Triple positive pixels make up most of the P2+ group.

**P2+ = P1+ minus unity of double positive pixels.

Supplementary Table 6. % CD68+ MC&M subtypes (**Figure 4C**)

		Pan 1 n=6	Pan 2 n=8	Pan 3 n=9	Pros 1 n=9	Pros 2 n=7	Pros 3 n=9	Kid 1 n=8	Kid 2 n=10	Kid 3 n=10
*single_CD68	Avg	85.680	31.772	40.283	68.954	27.189	40.738	58.854	36.668	23.244
	StDev	9.021	19.086	11.153	11.626	15.177	14.008	8.662	13.460	14.922
CD68_CD163	Avg	13.626	35.309	51.452	23.040	38.102	51.116	30.315	50.061	68.138
	StDev	8.834	11.465	15.973	10.343	10.638	11.459	8.699	14.393	19.047
CD68_CD11b	Avg	0.223	2.215	0.736	5.150	1.767	0.311	0.470	1.770	2.243
	StDev	0.102	2.170	0.635	7.230	1.184	0.332	0.912	1.184	2.426
CD68_CD11c	Avg	0.082	1.122	2.957	0.391	0.706	0.730	13.013	4.661	2.219
	StDev	0.096	1.546	6.292	0.270	0.695	1.078	7.481	2.269	2.546
CD206_CD68	Avg	0.556	28.095	3.374	1.927	1.061	7.120	1.129	0.274	2.531
	StDev	0.444	19.046	3.620	2.079	0.802	7.968	1.221	0.188	1.935
CD206_CD68_CD163	Avg	0.106	23.127	8.337	3.654	29.101	17.887	2.012	1.631	6.943
	StDev	0.122	10.980	4.995	3.045	13.411	10.909	2.772	1.121	8.659
CD206_CD68_CD11b	Avg	0.000	3.484	0.086	0.067	6.443	0.017	0.013	0.030	0.026
	StDev	0.000	5.225	0.109	0.122	4.632	0.026	0.025	0.051	0.055
CD206_CD68_CD11c	Avg	0.014	1.056	0.198	0.096	1.069	0.796	0.196	0.167	2.247
	StDev	0.031	1.458	0.141	0.194	1.083	1.289	0.293	0.288	4.497
CD68_CD163_CD11b	Avg	0.041	2.422	0.272	0.441	5.955	0.034	0.004	0.648	0.003
	StDev	0.087	3.860	0.217	0.490	4.484	0.045	0.008	0.302	0.007
CD68_CD163_CD11c	Avg	0.003	1.131	0.978	0.142	0.331	0.432	3.537	5.848	14.971
	StDev	0.006	1.541	0.464	0.202	0.382	0.564	2.924	2.695	16.610
CD68_CD11b_CD11c	Avg	0.004	0.782	0.050	0.040	0.217	0.014	0.119	0.255	0.473
	StDev	0.008	1.765	0.082	0.044	0.203	0.022	0.248	0.153	0.813

*Numbers are the fraction of all CD68+ pixels

*** single, double and triple positive pixels are obtained separately from the original image tiles. Double and triple positive pixels are the unity of two or three antibody masks

**Supplementary Table 7.** % CD163+ MC&M subtypes (**Figure 4D**)										
		Pan1 n=6	Pan2 n=8	Pan3 n=9	Pros1 n=9	Pros2 n=7	Pros3 n=9	Kid1 n=8	Kid2 n=10	Kid 3 n=10
single_CD163	Avg	72.438	53.177	60.050	25.886	32.347	59.552	34.941	42.639	6.395
	StDev	8.847	20.093	11.485	7.114	19.991	19.900	12.625	9.695	3.973
CD206_CD163	Avg	0.061	19.659	2.539	0.979	26.320	13.723	1.758	0.993	11.544
	StDev	0.066	7.485	3.426	2.295	17.047	6.816	2.609	1.018	4.769
CD68_CD163	Avg	27.144	23.358	35.696	70.464	35.080	25.993	53.630	47.190	20.351
	StDev	8.889	13.235	10.809	10.435	9.772	15.040	11.608	6.094	5.261
CD163_CD11b	Avg	0.051	1.495	0.274	0.165	1.751	0.161	0.005	0.139	0.340
	StDev	0.042	1.460	0.214	0.288	1.687	0.181	0.012	0.080	0.478
CD163_CD11c	Avg	0.273	0.754	0.521	0.039	0.238	0.437	3.276	2.366	0.454
	StDev	0.360	1.011	0.335	0.037	0.269	0.628	1.576	1.199	0.407
CD206_CD68_CD163	Avg	0.237	17.702	6.318	9.237	28.015	12.034	3.877	1.401	2.273
	StDev	0.346	14.493	4.589	5.543	14.447	11.301	4.921	0.820	2.993
CD206_CD163_CD11b	Avg	0.000	2.170	0.047	0.082	6.343	0.014	0.001	0.023	0.000
	StDev	0.000	3.535	0.064	0.099	4.012	0.014	0.002	0.039	0.001
CD206_CD163_CD11c	Avg	0.005	0.682	0.114	0.068	0.530	0.434	0.322	0.176	1.629
	StDev	0.012	1.044	0.109	0.133	0.417	0.567	0.498	0.262	4.280
CD68_CD163_CD11b	Avg	0.029	1.830	0.156	2.162	5.570	0.032	0.010	0.663	0.001
	StDev	0.057	3.008	0.094	3.483	3.695	0.055	0.025	0.338	0.002
CD68_CD163_CD11c	Avg	0.009	0.644	0.792	0.328	0.275	0.312	6.492	6.134	4.876
	StDev	0.021	0.800	0.504	0.273	0.200	0.368	4.158	4.161	5.821
CD163_CD11b_CD11c	Avg	0.000	0.623	0.007	0.023	0.178	0.004	0.005	0.087	0.000
	StDev	0.000	1.362	0.007	0.043	0.128	0.007	0.012	0.072	0.001

**Supplementary Table 8.** % of tissue in tile taken up by tumor, MC&M and T cell areas (**Figure 5**)									
% tumor in tile									
	Pan1 n=6	Pan2 n=8	Pan3 n=9	Pros1 n=9	Pros2 n=7	Pros3 n=9	Kid1 n=8	Kid2 n=10	Kid3 n=10
average	54.1	45.9	63.0	46.6	44.1	38.6	98.1	90.4	89.9
StDev	13.5	20.0	13.9	13.4	36.2	12.8	3.4	18.2	9.3
% MC&M area in tile									
average	5.2	8.8	8.9	16.0	12.6	8.0	4.5	12.2	3.6
StDev	1.7	4.1	2.6	11.0	2.4	1.5	1.9	4.2	1.6
% CD3 area in tile									
average	1.5	4.7	5.3	7.4	16.5	7.9	1.1	13.1	1.3
StDev	1.0	8.6	4.1	8.9	8.8	10.2	0.8	5.9	0.9

**Supplementary Table 9.** Density of individual MC&M subtypes in tumor area (**Figure 6B and C**)					
MC&M subtype	CD68*	CD163	CD11b	CD11c	All
Pancreas 1 n=6	6.13	1.77	0.04	0.04	7.4
	4.73	0.92	0.03	0.08	4.91
Pancreas 2 n=8	4.71	5.75	1.63	0.69	10.01
	3.25	3.34	1.61	0.69	5.69
Pancreas 3 n=9	5.43	8.20	0.51	0.76	11.62
	2.66	5.10	0.22	1.00	4.80
Prostate 1 n=9	13.91	1.29	0.70	0.11	14.47
	8.72	0.60	1.33	0.12	8.69
Prostate 2 n=7	7.01	6.79	3.00	0.92	13.63
	2.46	2.45	2.69	1.15	4.37
Prostate 3 n=9	1.31	2.25	0.08	0.37	3.42
	0.97	1.36	0.14	0.54	1.91
Kidney 1 n=8	2.36	1.24	0.10	1.73	4.33
	0.73	0.49	0.08	1.18	1.79
Kidney 2 n=10	8.26	9.23	0.88	1.64	13.53
	3.74	6.39	0.49	0.81	6.39
Kidney 3 n=10	0.93	3.26	0.30	0.18	3.94
	0.44	1.66	0.20	0.15	1.78

* CD68+ pixels in tumor area/tumor area

**Supplementary Table 10.** % of tumor M-mask (**Figure 6D**)				
MC&M subtype	CD68*	CD163	CD11b	CD11c
Pancreas 1 n=6**	75.61	27.73	0.57	0.71
	15.43	17.22	0.39	1.29
Pancreas 2 n=8	41.71	60.85	12.07	6.32
	20.28	16.13	10.32	7.56
Pancreas 3 n=9	46.83	66.94	5.31	8.26
	15.50	19.75	4.06	10.85
Prostate 1 n=9	93.67	12.36	4.81	1.29
	10.35	11.29	7.56	1.75
Prostate 2 n=7	51.54	51.94	20.09	6.51
	11.08	15.15	14.24	7.85
Prostate 3 n=9	35.06	68.68	3.77	7.91
	24.32	21.07	8.64	10.64
Kidney 1 n=8	59.83	29.98	2.44	34.49
	14.28	8.55	1.45	16.21
Kidney 2 n=10	61.77	63.37	7.05	12.40
	7.44	13.90	4.68	4.97
Kidney 3 n=10	25.09	80.25	9.11	4.99
	7.31	11.89	5.32	3.70

*total CD68+ pixels under tumor / total M-mask under tumor (average of tiles)
** n = number of tiles from which the average was calculated

Supplementary Table 11. Density of MC&M in tumor : density of MC&M in stroma (**Figure 6E**)

MC/M subtype		CD68	CD163	CD11b	CD11c
Pancreas 1 n=6**	Average	4.387*	1.483	0.422	0.724
	StDev	1.816	0.688	0.100	0.650
Pancreas 2 n=8	Average	2.887	1.698	1.447	1.322
	StDev	1.361	0.702	1.336	1.355
Pancreas 3 n=9	Average	3.829	3.169	1.333	1.701
	StDev	3.073	1.516	1.091	1.565
Prostate 1 n=9	Average	1.390	0.480	2.066	8.319
	StDev	1.423	0.705	5.418	17.625
Prostate 2 n=7	Average	1.058	0.816	1.315	4.063
	StDev	0.677	0.555	1.073	3.600
Prostate 3 n=9	Average	0.413	0.297	1.851	1.916
	StDev	0.321	0.198	1.118	1.022
Kidney 1 n=8	Average	1.254	0.213	1.141	1.897
	StDev	3.032	0.552	3.016	4.208
Kidney 2 n=10	Average	7.501	2.396	6.359	12.735
	StDev	5.822	2.575	10.397	19.585
Kidney 3 n=10	Average	80.519	77.170	4.095	5.846
	StDev	230.490	134.267	8.402	7.864

*macrophage pixels in tumor area/tumor area : macrophage pixels in stromal area/stromal area
pixels in stroma are calculated by subtracting the tumor pixels from the pixels in the tile
the average of n tiles is shown
**n = number of tiles per case

**Supplementary Table 12.** MC&M subtype distance from tumor **(Figure 6F)**							
**CD68**							
Distance (microns)	0	0-20	20-40	40-60	60-120	120-180	180-240
Pan n=23	11.339*	80.353	7.401	0.753	0.153	0.000	0.000
StDev	4.945	5.638	4.894	0.557	0.111	0.000	
Prostate n=25	16.578	62.728	14.396	3.165	2.912	0.222	0.000
StDev	9.947	4.736	7.735	3.291	4.118	0.314	
Average	13.959	71.541	10.899	1.959	1.533	0.111	0.000
**CD163**							
Distance (microns)	0	0-20	20-40	40-60	60-120	120-180	180-240
Pan n=23	8.401	70.661	16.676	2.851	1.240	0.171	0.000
StDev	3.080	11.129	9.757	2.382	1.194	0.242	
Prostate n=25	10.216	68.303	16.949	2.740	1.677	0.116	0.000
StDev	2.663	3.373	2.977	1.315	1.407	0.164	
Average	9.309	69.482	16.813	2.795	1.458	0.143	0.000
**CD11b**							
Distance (microns)	0	0-20	20-40	40-60	60-120	120-180	180-240
Pan n=23	2.620	43.668	23.047	11.577	13.617	3.865	1.606
StDev	1.699	13.566	5.247	3.272	9.177	2.898	2.005
Prostate n=25	6.528	37.500	22.610	10.495	14.336	3.983	1.705
StDev	4.286	16.071	3.648	6.504	10.183	3.871	2.253
Average	4.574	40.584	22.829	11.036	13.976	3.924	1.655
**CD11c**							
Distance (microns)	0	0-20	20-40	40-60	60-120	120-180	180-240
Pan n=23	2.064	31.180	27.328	16.131	17.808	4.342	1.147
StDev	1.722	13.356	6.574	1.788	12.760	5.222	1.480
Prostate n=25	6.996	40.792	23.695	10.809	14.949	2.155	0.605
StDev	3.945	10.674	6.618	5.654	9.967	1.535	0.855
Average	4.530	35.986	25.512	13.470	16.378	3.249	0.876

*percent of macrophage-tumor distances between 0 and 20 microns

**Supplementary Table 13.** MC&M subtype – T cell overlap (**Figures 7B +7C**)						
MC/M subtypes		CD68	CD163	CD11b	CD11c	Total MC/M
Pan 1 n=6**	Average	16.36*	20.15	0.76	0.90	29.89
	StDev	9.20	8.13	1.04	1.57	13.68
Pan 2 n=8	Average	6.88	13.78	2.92	1.22	20.17
	StDev	4.98	6.88	2.62	1.03	8.56
Pan 3 n=9	Average	8.91	14.01	1.55	1.90	19.86
	StDev	4.27	9.89	1.22	2.46	8.63
Pros 1 n=9	Average	31.03	20.72	1.09	0.30	34.00
	StDev	18.74	20.82	2.59	0.35	21.29
Pros 2 n=7	Average	19.37	22.19	4.02	0.52	31.98
	StDev	13.20	12.90	2.94	0.44	15.26
Pros 3 n=9	Average	20.21	43.14	0.13	0.60	50.82
	StDev	12.85	24.43	0.12	0.75	24.33
Kid 1 n=8	Average	29.39	20.42	1.85	6.87	36.71
	StDev	19.37	16.53	2.42	4.47	20.79
Kid 2 n=10	Average	10.79	11.98	1.85	2.57	18.48
	StDev	3.46	6.08	2.45	1.14	5.23
Kid 3 n=10	Average	7.08	15.37	2.88	0.56	18.91
	StDev	7.14	13.49	2.76	0.56	12.75

*the average percentage of CD68+ pixels in case that overlap with CD3+ pixels and the standard deviation across the tiles of the case.
**tiles in case

**Supplementary Table 14.** % MC&M overlapping with T cells (**Figure 7D**)				
MC&M subtypes	CD68	CD163	CD11b	CD11c
Pan 1 n=6	49.532*	71.208	2.827	1.852
Pan 2 n=8	30.214	68.956	12.630	7.789
Pan 3 n=9	45.460	63.238	8.517	13.013
Pros 1 n=9	91.199	55.661	3.242	1.266
Pros 2 n=7	57.627	67.235	11.714	1.533
Pros 3 n=9	44.762	81.545	0.572	2.384
Kid 1 n=8	73.954	47.346	5.202	23.122
Kid 2 n=10	58.793	62.958	9.591	14.177
Kid 3 n=10	31.222	74.242	17.890	4.044

*percentage CD68+ pixels of total MC/M-mask

**Supplementary Table 15.** Mean distance of MC&M subtypes to T cells (microns) (**Figure 7E**)				
MC&M subtypes	CD68	CD163	CD11b	CD11c
Pan 1 n=6	27.008	19.863	250.398	52.269
	2.638	2.536	19.509	6.042
Pan 2 n=8	6.140	22.679	76.663	215.053
	2.519	6.490	14.832	31.777
Pan 3 n=9	8.636	19.677	86.413	31.112
	1.026	1.927	4.913	3.426
Pros 1 n=9	4.536	16.828	36.580	68.218
	0.823	1.681	3.055	6.223
Pros 2 n=7	11.016	9.913	12.527	29.659
	1.037	1.029	1.066	1.903
Pros 3 n=9	10.141	7.023	79.456	27.811
	1.209	0.730	5.349	3.129
Kid 1 n=8	43.165	7.075	9.408	75.83
	2.975	0.611	0.731	3.751
Kid 2 n=10	13.49	30.65	190.62	7.67
	4.477	10.367	24.932	2.903
Kid 3 n=10	31.176	14.733	95.772	87.644
	4.281	2.900	10.078	9.824
